# Impairment of peroxisomal APX and CAT activities increases protection of photosynthesis under oxidative stress

**DOI:** 10.1093/jxb/ery354

**Published:** 2018-10-12

**Authors:** Rachel H V Sousa, Fabricio E L Carvalho, Yugo Lima-Melo, Vicente T C B Alencar, Danilo M Daloso, Marcia Margis-Pinheiro, Setsuko Komatsu, Joaquim A G Silveira

**Affiliations:** 1 Department of Biochemistry and Molecular Biology, Federal University of Ceará, Fortaleza, Ceará, Brazil; 2 Molecular Plant Biology, Department of Biochemistry, University of Turku, Turku, Finland; 3 Department of Genetics, Federal University of Rio Grande do Sul, Porto Alegre, Rio Grande do Sul, Brazil; 4 Graduate School of Life and Environmental Sciences, University of Tsukuba, Tsukuba, Japan

**Keywords:** Ascorbate peroxidase, H_2_O_2_ signalling, oxidative stress, photosynthetic efficiency, proteomics, redox metabolism

## Abstract

Retrograde signalling pathways that are triggered by changes in cellular redox homeostasis remain poorly understood. Transformed rice plants that are deficient in peroxisomal ascorbate peroxidase APX4 (*OsAPX4*-RNAi) are known to exhibit more effective protection of photosynthesis against oxidative stress than controls when catalase (CAT) is inhibited, but the mechanisms involved have not been characterized. An in-depth physiological and proteomics analysis was therefore performed on *OsAPX4*-RNAi CAT-inhibited rice plants. Loss of APX4 function led to an increased abundance of several proteins that are involved in essential metabolic pathways, possibly as a result of increased tissue H_2_O_2_ levels. Higher photosynthetic activities observed in the *OsAPX4*-RNAi plants under CAT inhibition were accompanied by higher levels of Rubisco, higher maximum rates of Rubisco carboxylation, and increased photochemical efficiencies, together with large increases in photosynthesis-related proteins. Large increases were also observed in the levels of proteins involved in the ascorbate/glutathione cycle and in other antioxidant-related pathways, and these changes may be important in the protection of photosynthesis in the *OsAPX4*-RNAi plants. Large increases in the abundance of proteins localized in the nuclei and mitochondria were also observed, together with increased levels of proteins involved in important cellular pathways, particularly protein translation. Taken together, the results show that *OsAPX4*-RNAi plants exhibit significant metabolic reprogramming, which incorporates a more effective antioxidant response to protect photosynthesis under conditions of impaired CAT activity.

## Teaser

Proteomics and physiological approaches reveal that peroxisomal APX silencing and CAT inhibition trigger H_2_O_2_-signalling for up-regulation of antioxidant proteins to protect the rice photosynthetic apparatus under oxidative stresss.

## Introduction

Plant peroxisomes are the most important cellular site for hydrogen peroxide (H_2_O_2_) production in C_3_ plants exposed to light. Several studies have shown that redox changes in these organelles are able to affect metabolic regulation in other cellular compartments by cross-talk mechanisms (Nyathi and Baker, 2006; Sewelam *et al.*, 2014; Corpas, 2015). The majority of these findings have been achieved by using plants deficient in catalase (CAT, EC 1.11.1.6), indicating that such responses are associated with photorespiratory H_2_O_2_ accumulation and downstream oxidative signalling events (Willekens *et al.*, 1997; Rizhsky *et al.*, 2002; Vandenabeele *et al.*, 2004; Vanderauwera *et al.*, 2011; Han *et al.*, 2013; Rahantaniaina *et al.*, 2017). Transcriptomic and proteomic analyses have revealed that alterations in peroxisomal metabolism are able to trigger several transcriptional and translational modifications that affect a number of metabolic pathways, including changes in various ribosomal and heat-shock proteins (Vandenabeele *et al.*, 2004; Vanderauwera *et al.*, 2005, 2011). However, the physiological significance of those alterations and how plants adjust their metabolism under CAT inhibition remain unclear.

Our understanding of how a deficiency in CAT activity and H_2_O_2_ signalling can affect such metabolic pathways is still incomplete (Sousa *et al.*, 2015). This is a significant problem because the majority of common abiotic stresses can enhance the photorespiratory production of H_2_O_2_ and some of them are capable of inducing CAT degradation, and hence decreasing its activity (Hertwig *et al.*, 1992; Polidoros and Scandalios, 1997; Voss *et al.*, 2013). In addition, even less is known about which other peroxisomal peroxidases are capable of overlapping with CAT activity under such stressful conditions. Peroxisomal isoforms of ascorbate peroxidase (APX, EC 1.11.1.11) are generally regarded as the most important plant peroxidases related to compensation and/or supplementation of CAT activity (Yamaguchi *et al.*, 1995; Mullen *et al.*, 1999; Wang *et al.*, 1999; Kavitha *et al.*, 2008; Xu *et al.*, 2008); however, there is no consensus about this, since some studies employing Arabidopsis and rice plants deficient in peroxisomal APX (pAPX) have suggested only minor importance for these proteins as H_2_O_2_ scavengers (Narendra *et al.*, 2006; Sousa *et al.*, 2015). It therefore remains unclear whether the different proteins of the peroxisomal redox network can compensate for each other.

CAT and pAPX have very contrasting *K*_M_ values for H_2_O_2_, which suggests that these peroxidases could display complementary metabolic roles in the peroxisomes (Mhamdi *et al.*, 2012). Whilst pAPX may be important for fine-tuning under low concentrations of H_2_O_2_, CAT seems to be a key factor for scavenging when H_2_O_2_ concentrations are high (Corpas, 2015). There is evidence to suggest that pAPX isoforms are externally bound to peroxisome membranes, with the catalytic sites facing the cytosol (Yamaguchi *et al.*, 1995; Kavitha *et al.*, 2008; Ribeiro *et al.*, 2017). This could favour the establishment of a signalling interface between the peroxisomes and the cytosol, given that pAPX activity may contribute to the balance of H_2_O_2_ content between the two. Recent studies have demonstrated that peroxisomal H_2_O_2_ might migrate to the cytosol and other neighboring organelles via membranes and trigger specific signals for gene expression (Mubarakshina *et al.*, 2010; Corpas, 2015; Exposito-Rodriguez *et al.*, 2017). H_2_O_2_ specifically produced in peroxisomes and chloroplasts is known to be able to trigger differential gene expression by retrograde signalling (Sewelam *et al.*, 2014). These findings suggest that in addition to the H_2_O_2_ localization, the antioxidant system involved with its scavenging is also important for the signalling response (Foyer, 2018).

Deficiency in cytosolic APX1 leads to increased chloroplastic H_2_O_2_ levels that are associated with down-regulation of both the chloroplastic APX isoforms (stromal and thylakoidal), indicating that there is a cross-talk mechanism between the chloroplast and cytosol (Davletova *et al.*, 2005). In addition, tobacco and Arabidopsis *cat2 apx1* double-mutants are more resistant to paraquat-induced oxidative stress than single-mutants (Rizhsky *et al.*, 2002; Vanderauwera *et al.*, 2011). These results are in agreement with studies reported by our group that employed rice plants deficient in peroxisomal or cytosolic APX that were subjected to pharmacological CAT inhibition (Sousa *et al.*, 2015; Bonifacio *et al.*, 2016). The Arabidopsis *cat2 apx1* double-mutant displays specific reactive oxygen species (ROS) signalling triggered by simultaneous CAT and APX deficiency, which contributes to a more effective DNA repair system associated with control of the cell cycle and suppression of the plant cell-death pathway (Vanderauwera *et al.*, 2011). Taken together, these results serve to emphasize that physiological responses generated by signalling involving H_2_O_2_ are very intricate since they are dependent on multifaceted metabolic and gene networks. Thus, to understand this complex and highly compartmentalized signalling network, studies using multi-transformation approaches or combining plant transformation with pharmacological inhibition are necessary, and this is the approach adopted here.

Given that CAT knockdown/knockout has severe consequences for plant growth and development (Chamnongpol *et al.*, 1996; Willekens *et al.*, 1997), the pharmacological inhibition of this enzyme through the use of a specific inhibitor such as 3-amino-1,2,4-triazole (3-AT) is important to avoid the pleotropic effects caused by the constitutive depletion of this enzyme. Furthermore, this strategy provides great opportunities to investigate the effect of CAT inhibition at different spatial-temporal scales. Indeed, 3-AT has been widely used in plant biology as an efficient CAT-inhibitor, inducing only small side effects in redox metabolism (Sousa *et al.*, 2015; Rahantaniaina *et al.*, 2017). Taking this into account, we have investigated the effects of simultaneous deficiencies of APX and CAT by exposing cytosolic or peroxisomal APX-silenced rice plants to 3-AT. These plants display lower ROS accumulation and higher photosynthetic resilience in comparison to CAT-inhibited non-transformed plants (Sousa *et al.*, 2015; Bonifacio *et al.*, 2016), but the underlying mechanisms remain unknown.

In order to explain these previous results, in this study we tested the hypothesis that rice plants simultaneously deficient in pAPX and CAT might trigger a differential retrograde H_2_O_2_ signalling mechanism that is able to induce effective antioxidant protection for photosynthesis. We performed an integrated approach using silencing of peroxisomal APX4 combined with pharmacological inhibition of CAT by 3-AT together with an associated high-throughput proteomic analysis and *in vivo* measurements of photosynthesis. The results corroborate our previous findings and provide evidence that peroxisomal APX deficiency combined with CAT inhibition trigger a strong increase in several important proteins related to crucial metabolic processes. Accumulation of proteins belonging to the ascorbate/glutathione cycle targeted to the cytosol and chloroplasts, together with accumulation of other antioxidants and protective proteins, were important for maintaining higher photosynthetic rates when compared to non-transformed plants. The physiological significance of these results for photosynthetic resilience and H_2_O_2_ signalling under oxidative stress conditions is discussed.

## Material and methods

### Growth conditions and treatments

RNAi knockdown plants of rice (*Oryza sativa* ssp. *japonica* cv. Nipponbare) deficient in peroxisomal ascorbate peroxidase 4 (*OsAPX4*-RNAi) were obtained as previously described (Ribeiro *et al.*, 2017). Non-transformed (NT) and *OsAPX4*-RNAi seedlings were transferred to 2-l pots containing Hoagland–Arnon nutrient solution (Hoagland and Arnon, 1950) and placed in a controlled growth chamber for 40 d (day/night mean temperature 29/24 °C, mean relative humidity 68%, photoperiod 12 h, and 400 µmol photons m^−2^ s^−1^ PPFD).

For the CAT inhibition experiment, 40-d-old NT and *OsAPX4*-RNAi plants were sprayed with 10 mM 3-amino-1,2,4-triazole (3-AT) dissolved in 10 mM HEPES buffer (pH 6.5) containing 1.5 mM CaCl_2_ and 0.1% Triton X-100. Leaves were sprayed once with 50 ml of 3-AT or a mock solution at the beginning of the 12-h dark period. Gas exchange measurements and leaf sampling for the proteomic analysis were then performed at the end of the following 12-h light period. For the short-term CAT inhibition and H_2_O_2_ accumulation experiment, leaves of 40-d-old NT and *OsAPX4*-RNAi plants were sprayed once with 50 ml 3-AT or mock control solutions as described above, and leaf sampling was performed at 0, 0.5, 1.5, and 2.5 h after treatment. During this time-course experiment, plants were kept in the growth chamber under the conditions described above. To investigate the consequences of CAT and APX4 deficiencies on PSII activity, 40-d-old NT and *OsAPX4*-RNAi plants were sprayed with 10 mM 3-AT or mock control solutions and kept at the growth-chamber light intensity for 1 h (400 µmol photons m^−2^ s^−1^). The plants were then exposed to high light (1000 µmol photons m^−2^ s^−1^) for 1 h to increase photo-oxidative stress, and *in vivo* chlorophyll *a* fluorescence measurements were then performed.

### Gas exchange measurements

The relationship between intercellular CO_2_ partial pressure and photosynthesis (*A*/*C*_i_ curve) was determined using a portable infrared gas analyser system, equipped with an LED source and a leaf chamber (IRGA LI-6400XT, LI-COR, Lincoln, NE, USA). *C*_i_ was varied between 50 and 1800 ppm CO_2_ and the photosynthetically active radiation (PAR) was set as 1000 μmol photons m^−2^ s^−1^. The maximum Rubisco carboxylation rate (*V*_cmax_) and maximum photosynthetic electron transport rate (*J*_max_) were calculated from the *A*/*C*_i_ curve (Sharkey *et al.*, 2007). Measurements were carried out on the newest fully expanded leaf.

### Chlorophyll *a* fluorescence

The saturation pulse method (Schreiber *et al.*, 1995) was used to evaluate the *in vivo* PSII activity using a Dual-PAM-100 fluorometer (Walz, Germany). The newest fully expanded leaf was detached from the plant and dark-acclimated for 30 min in order to assess the maximum quantum yield of PSII, which was estimated as *F*_v_/*F*_m_ = (*F*_m_–*F*_o_)/*F*_m_, where *F*_o_ and *F*_m_ represent the minimal and the maximum fluorescence after a saturation pulse in the dark, respectively. The saturation pulse intensity was 8000 μmol photons m^−2^ s^−1^ and the duration was 0.6 s. The leaves were then exposed to actinic light (500 μmol photons m^−2^ s^−1^) and a second saturation pulse was applied after 3 min in order to assess the actual quantum yield, which was estimated as Y(II) = (*F*′_m_-*F*_s_)/*F*′_m_, where *F*′_m_ and *F*_s_ are the maximum and steady-state fluorescence in the light, respectively. The electron transport ratio from PSII was estimated as ETR(II) = Y(II)×500 × 0.5 × 0.8, assuming that 80% of incident light reached the PSII antennas and an equal energy distribution between PSII and PSI had occurred.

### Western blotting

The Rubisco large subunit (rbcL) was identified in total soluble extracts by immunoblotting. The central portion of the newest fully expanded leaf was used for extraction, which was performed using liquid N_2_ in the presence of 100 mM phosphate buffer (pH 7.5) containing 2 mM EDTA and 1 mM ascorbate. SDS-PAGE was performed with 10 μg proteins from the total soluble extract. After electrophoresis, the proteins were electrophoretically transferred to a nitrocellulose membrane (Towbin *et al.*, 1979), and detection was performed using specific polyclonal antibodies against rbcL (AS03037, Agrisera, Vännäs, Sweden). The tagged protein was detected by luminescence, through the reaction between the second antibody bound to the target protein and the ECL reagent (GE Healthcare, ref. RPN2106). Membrane images were captured and the different bands were quantified using a SmartView Pro 1200 Image System (Major Science, Taiwan).

### Catalase activity

Catalase activity was assayed from total soluble extracts from the central portion of the newest fully expanded leaf by following the oxidation of H_2_O_2_ at 240 nm over a 300-s period at 25 °C in the presence of 50 mM potassium phosphate buffer (pH 7.0) containing 20 mM H_2_O_2_ (Havir and McHale, 1987). CAT activity was calculated according to the H_2_O_2_ molar extinction coefficient (36 M^−1^ cm^−1^) and the results were expressed as µmol H_2_O_2_ mg^−1^ protein min^−1^. The total protein concentration was quantified according to Bradford (1976).

### Protein extraction, digestion, and desalting for proteomic analysis

Approximately 700 mg of material from the central portion of the newest fully expanded leaf from each plant were ground to a powder in liquid N_2_, frozen, and dried by lyophilization. The extracted proteins were precipitated in 10% trichloroacetic acid and 0.07% 2-mercaptoethanol in acetone, and resuspended in a lysis buffer containing 8 M urea, 2 M thiourea, 5% CHAPS, and 2 mM tributylphosphine. The protein concentrations were determined according to Bradford (1976) with BSA as the standard. The samples were purified with methanol and chloroform to remove detergent and centrifuged at 20000 *g* for 10 min to achieve phase separation. The upper phase was discarded and methanol was added to the lower phase. The solutions were again centrifuged at 20000 *g* for 10 min and the resulting pellets were dried, then reduced with 25 mM dithiothreitol, and alkylated with 30 mM iodoacetamide. The alkylated proteins were digested with trypsin and lysyl endopeptidase (Wako, Osaka, Japan) at a 1:100 enzyme:protein ratio at 37 °C for 16 h in the dark. Peptides were acidified with 20% formic acid (pH<3) and desalted with a C18-pipette tip (Nikkyo Technos, Tokyo, Japan). The samples were analysed by nano-liquid chromatography with tandem mass spectrometry (LC-MS/MS), as described below.

### Protein identification using nano-LC-MS/MS

The peptide samples were separated using an Ultimate 3000 nano-LC system (Dionex, Germering, Germany), and the peptide ions were detected using a LTQ Orbitrap Discovery MS nanospray (ThermoFisher Scientific, Waltham, MA, USA) with data-dependent acquisition mode with the Xcalibur software (version 2.1, ThermoFisher Scientific). The peptide samples were loaded onto a C18 PepMap trap column (300 µm I.D. × 5 mm, ThermoFisher Scientific) equilibrated with 0.1% formic acid and eluted from the trap column with a linear acetonitrile gradient in 0.1% formic acid at a flow rate of 200 nl min^−1^. The eluted peptides were loaded and separated on a C18 capillary tip column (75 µm I.D. × 120 mm, NikkyoTechnos, Tokyo, Japan) with a spray voltage of 1.5 kV. Full-scan mass spectra were acquired in the Orbitrap MS over 400–1500 *m*/*z* with a resolution of 30000. The top 10 most intense precursor ions were selected for collision-induced fragmentation in the linear ion trap at a normalized collision energy of 35%. Dynamic exclusion was employed within 90 s to prevent the repetitive selection of peptides (Zhang *et al.*, 2009).

### Data acquisition by MS analysis

Identification of proteins was performed using the Mascot Server (version 2.4.1., http://www.matrixscience.com/server.html) with a rice protein database (50253 sequences and 15266515 residues) obtained from The Rice Annotation Project Database (RAP-DB, http://rapdb.dna.affrc.go.jp), including protein sequences supported by FL-cDNA and EST data (IRGSP-1.0_protein_ 2013-4-24) and protein sequences predicted computationally (IRGSP-1.0_ predicted protein_2013-3-9). The Proteome Discoverer software (version 1.4, ThermoFisher Scientific) was used to process the acquired raw data files. For the Mascot searches, the carbamidomethylation of cysteine was set as a fixed modification, and oxidation of methionine was set as a variable modification. Trypsin was specified as the proteolytic enzyme and one missed cleavage was allowed. Peptide mass tolerance was set at 10 ppm, fragment mass tolerance at 0.5 Da, and the peptide charge at +2, +3, and +4. An automatic decoy database search was performed as part of the search. The Mascot results were filtered with the Percolator function to improve the accuracy and sensitivity of peptide identification. The acquired Mascot results were imported to the SIEVE software (version 2.1, ThermoFisher Scientific).

### Differential analysis of proteins using MS data

The commercial label-free quantification package SIEVE was used for the differential analysis of relative abundances of peptides and proteins between samples. The chromatographic peaks detected by MS were aligned, and the peptide peaks were detected as a frame on all parent ions scanned by MS/MS using 5 min of frame time width and 10 ppm of frame *m*/*z* width. Chromatographic peak areas within a frame were compared for each sample, and the ratios between samples in a frame were determined. The frames detected in the MS/MS scan were matched to the imported Mascot results. The peptide ratio between samples was determined from the variance-weighted average of the ratios in frames that matched the peptides in the MS/MS spectrum. The ratios of peptides were further integrated to determine the ratio of the corresponding proteins. In the differential analysis of protein abundance, the total ion current was used for normalization. The minimum requirement for identification of a protein was two matched peptides. Significant changes in the abundance of proteins between samples were analysed (*P*≤0.05).

### Functional analysis

Functional analysis of the identified proteins was performed using MapMan bin codes (http://mapman.gabipd.org/) according to Usadel *et al.*, (2005).

### Statistical analyses

Experiments were carried out in a completely randomized design with four replicates, each one represented by an individual pot containing two plants. Data were analysed by ANOVA and means were compared by Tukey’s test at the *P*<0.05 confidence level.

## Results

### RNAi-silencing down-regulates both *OsApx3* and *OsApx4* gene expression and strongly decreases APX4 abundance whereas 3-AT induces fast inhibition of CAT

**Fig. 1.**
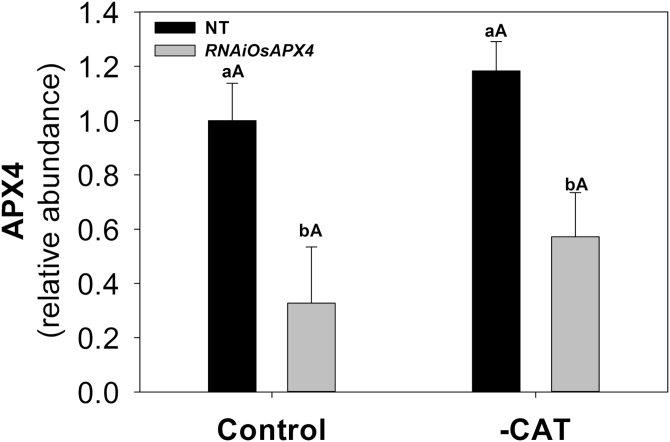
Relative abundance of ascorbate peroxidase 4 (APX4) in leaves of non-transformed (NT) and *OsAPX4*-RNAi rice plants (control) and plants treated with the catalase-inhibitor 3-AT (–CAT). The relative amount of APX4 was obtained by differential analysis of proteins using MS data, as described in the Methods. Data are means (±SD) of four replicates. Capital letters indicate significant differences between treatments (control and –CAT) within the same genotype and lower-case letters represent significant differences between genotypes (NT and *OsAPX4*-RNAi) within each treatment (*P*≤0.05).

**Fig. 2.**
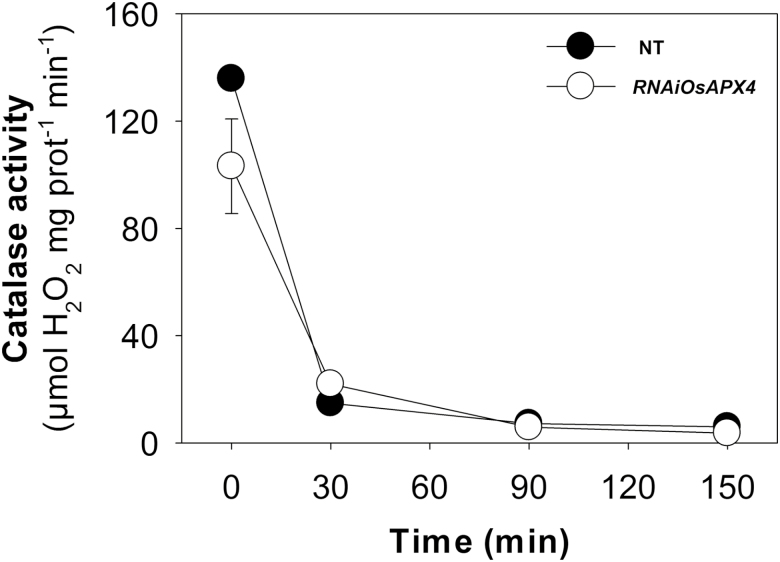
Catalase activity in leaves of non-transformed (NT) and *OsAPX4*-RNAi rice plants following treatment with 10 mM of the CAT-inhibitor 3-AT. Data are means (±SD) of four replicates.

As previously reported, the RNAi-silenced rice lines that we used do not display phenotypic changes at the morphological and physiological levels during the vegetative phase despite both peroxisomal *OsAPX3* and *OsAPX4* transcript levels being significantly decreased after knockdown (Sousa *et al.*, 2015; Ribeiro *et al.*, 2017). The silencing resulted in a decrease in abundance of the APX4 protein by about 60% in the *OsAPX4*-RNAi line compared with non-transformed (NT) controls, which was reduced to a 40% decrease when plants were exposed to 3-AT (Fig. 1). These changes in APX4 protein abundance are associated with unchanged cytosolic APX activity and decreased chloroplastic APX activity in silenced plants (Ribeiro *et al.*, 2017). After NT and *OsAPX4*-RNAi plants were treated with 10 mM 3-AT, rapid inhibition of CAT was observed, with almost 100% inhibition occurring after 90 min (Fig. 2). This treatment regime provided simultaneous deficiencies of pAPX and CAT in the transgenic plants and CAT deficiency alone in the NT plants for about 24 h, which was enough to affect the translation mechanisms and consequently the proteomic profiles. At 24 h after treatment of the 40-d-old mature plants with 3-AT neither the NT nor the transformed plants exhibited any visual symptoms of toxicity (Supplementary Fig. S1 at *JXB* online), indicating that the dose employed did not cause generalized side-effects, in accordance with our previous results (Bonifacio *et al.*, 2016). In order to determine whether the effects on photosynthesis induced by 3-AT were exclusively due to CAT inhibition or were caused by indirect mechanisms, an experiment was carried out under non-photorespiratory conditions (3% CO_2_). Under these conditions, both the NT and *OsAPX4*-RNAi plants displayed similar rates of photosynthesis after treatment with 3-AT, which were comparable to their respective controls (data not shown). These results indicated that the effects of 3-AT were essentially related to CAT inhibition and other downstream oxidative mechanisms, and that there was no direct effect on rice photosynthetic activity.

### *OsAPX4*-RNAi lines display higher photosynthetic capacity than NT plants after CAT inhibition

**Fig. 3.**
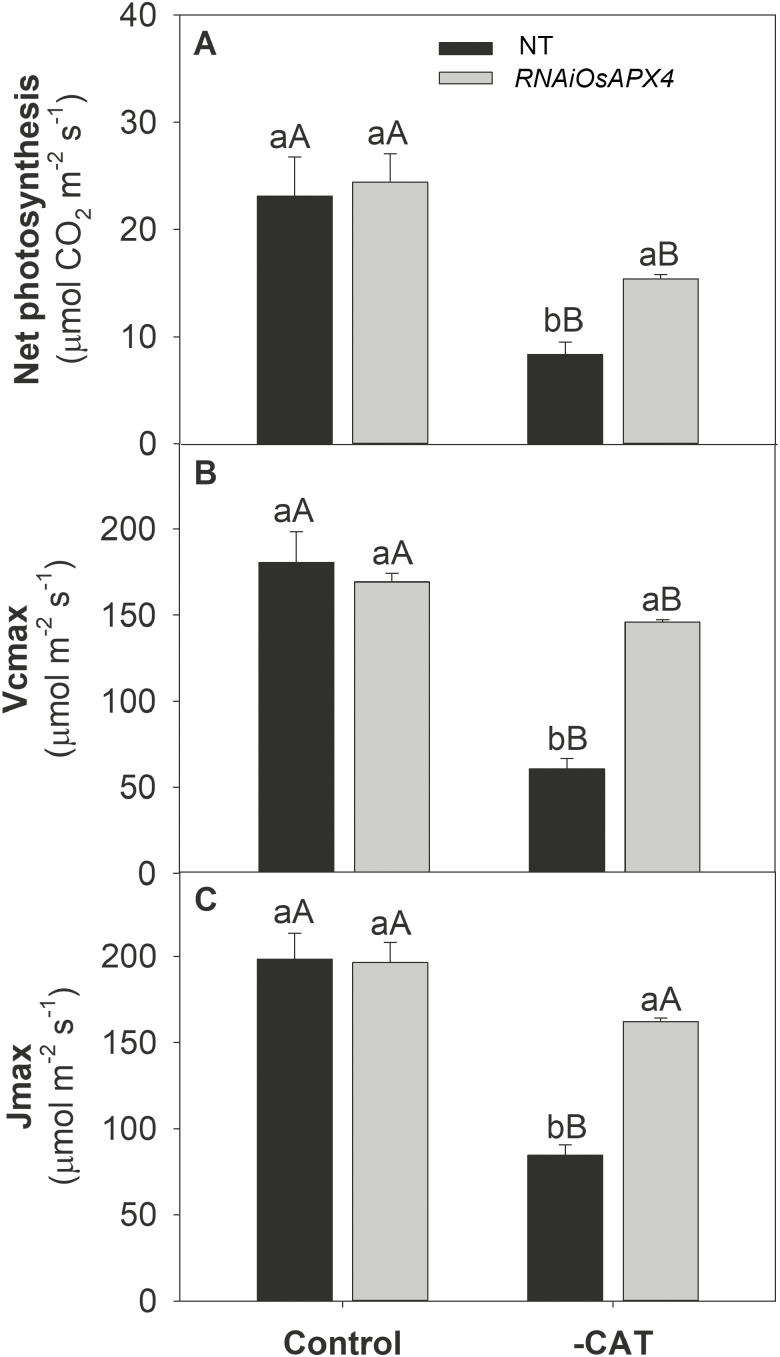
(A) Net photosynthesis, (B) maximum Rubisco carboxylation rate (*V*_cmax_), and (C) maximum photosynthetic electron transport rate (*J*_max_) in leaves of non-transformed (NT) and *OsAPX4*-RNAi rice plants (control) and plants treated with 10 mM of the catalase-inhibitor 3-AT (–CAT). Data are means (±SD) of four replicates. Capital letters indicate significant differences between treatments (control and –CAT) within the same genotype and lower-case letters represent significant differences between genotypes (NT and *OsAPX4*-RNAi) within each treatment (*P*≤0.05).

**Fig. 4.**
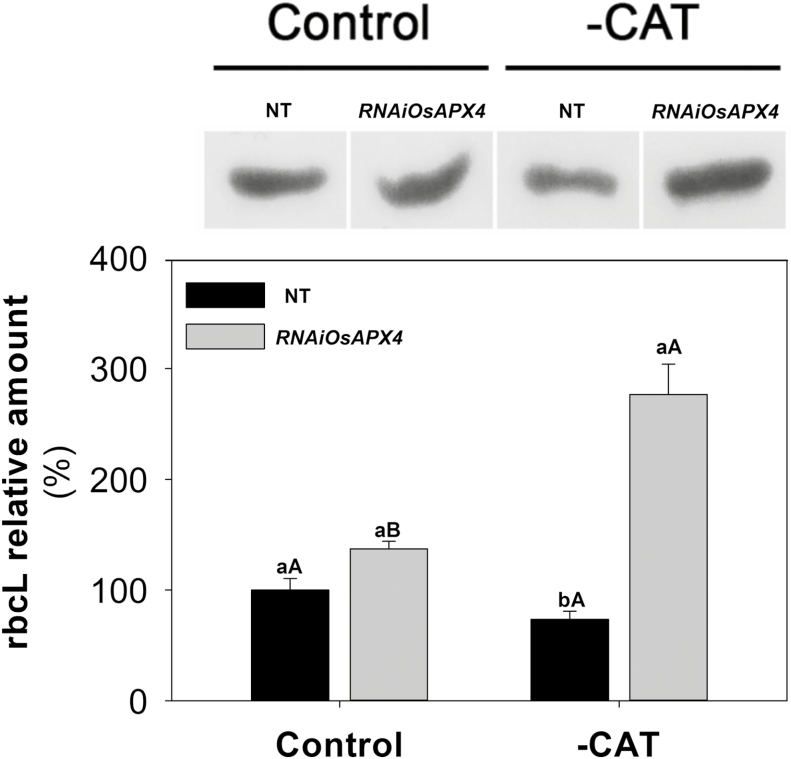
Western blots of the Rubisco large subunit (rbcL) in leaves of non-transformed (NT) and *OsAPX4*-RNAi rice plants (control) and plants treated with 10 mM of the catalase-inhibitor 3-AT (–CAT). For SDS-page, 10 µg of soluble protein was loaded in each lane. The membrane images were captured and the different bands were quantified using a SmartView Pro 1200 Image System. Data are means (±SD) of band quantifications from three independent runs.

**Fig. 5.**
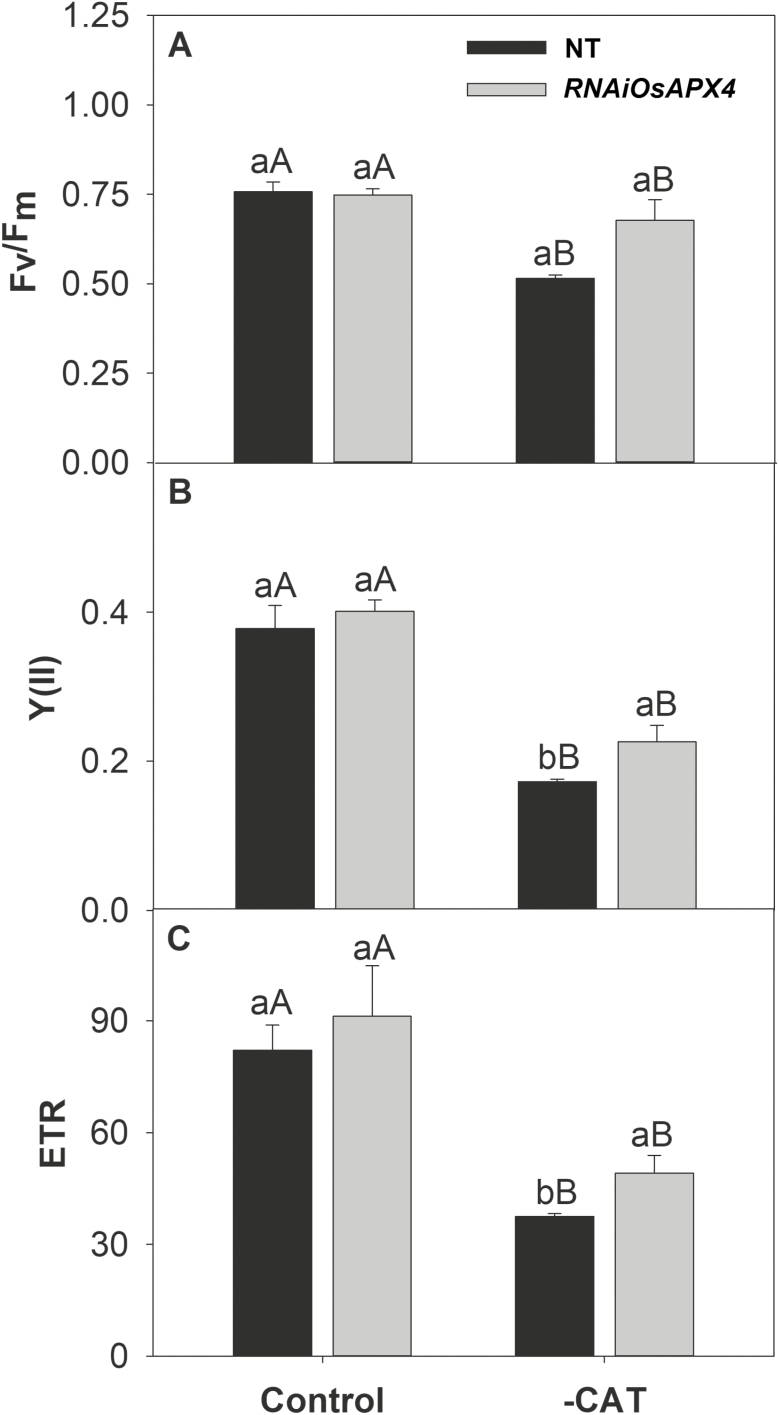
(A) Maximum quantum yield of PSII (*F*_v_/*F*_m_), (B) actual quantum yield of PSII, Y(II), and (C) electron transport rate (ETR) in leaves of non-transformed (NT) and *OsAPX4*-RNAi rice plants (control) and plants treated with 10 mM of the catalase-inhibitor 3-AT (–CAT). Data are means (±SD) of four replicates. Capital letters indicate significant differences between treatments (control and –CAT) within the same genotype and lower-case letters represent significant differences between genotypes (NT and *OsAPX4*-RNAi) within each treatment (*P*≤0.05).

To corroborate our previous study (Sousa *et al.*, 2015) and to calculate gas exchange parameters related to photosynthetic efficiency, *A*/*C*_i_ curves were constructed (Supplementary Fig. S2). The results indicated that both genotypes showed similar net photosynthesis (*A*) under normal growth conditions. CAT inhibition induced a significant reduction in *A* in both plant types, but the decrease was more prominent in NT than in the *OsAPX4*-RNAi line (63% and 37% reduction, respectively, compared to the respective controls at ambient CO_2_; Fig. 3A). The greater decrease in *A* was accompanied by strong reductions in the maximum Rubisco carboxylation rate (*V*_cmax_) and the maximum electron transport rate (*J*_max_), which decreased by 66% and 57% in NT, and 14% and 17% in *OsAPX4*-RNAi plants, respectively (Fig. 3B, C). The higher *V*_cmax_ and *A* exhibited by *OsAPX4*-RNAi plants were closely related to Rubisco abundance, which was significantly increased in leaves of *OsAPX4*-RNAi plants and decreased in those of NT when compared to their respective controls (Fig. 4). To determine whether the effects of 3-AT on gas exchange parameters were related to photochemical disturbances, PSII activity indicators were measured after a 1-h exposure to high light (1000 µmol photons m^−2^ s^−1^ PPFD), which was applied 1 h after treatment with 3-AT. The maximum quantum yield of PSII (*F*_v_/*F*_m_) in the NT and *OsAPX4*-RNAi plants was similar under normal growth conditions, but after exposure to 3-AT and high light, it was significantly decreased in the NT plants (Fig. 5A), indicating strong photoinhibition of PSII. These photochemical alterations were paralleled by changes in the actual quantum yield of PSII [Y(II)] and the electron transport rate of PSII (ETR), which decreased more in the NT plants (Fig. 5B, C).

### *OsAPX4*-RNAi plants have increased abundance in numerous proteins and CAT inhibition strongly enhances this response in several metabolic pathways

RNAi-silenced rice plants displayed increases in abundance of 211 different proteins involved in a number of biochemical pathways in comparison to NT plants (Supplementary Table S1 and Supplementary Fig. S3). *OsAPX4*-RNAi plants showed a notable increase in abundance of 636 proteins after CAT inhibition, whereas the corresponding number in NT plants was only 252. The increases in abundance of proteins in CAT-inhibited *OsAPX4*-RNAi plants in particular included metabolic processes related to photosynthesis (59), the TCA cycle (27), amino acids (37), biotic/abiotic stresses (33), redox pathways (32), and DNA/RNA (38) and protein metabolism (147) (Supplementary Fig. S3). The number of proteins with decreased abundance was very much lower than that of proteins with increased abundance across all treatments (Supplementary Fig. S4). In contrast to the increased proteins, the NT and *OsAPX4*-RNAi plants had similar results for the amount of decreased proteins under CAT inhibition (70 and 74, respectively). The decreased proteins were related to a relatively restricted number of metabolic processes compared to those that were increased, especially in *OsAPX4*-RNAi plants (Supplementary Fig. S4).

**Fig. 6.**
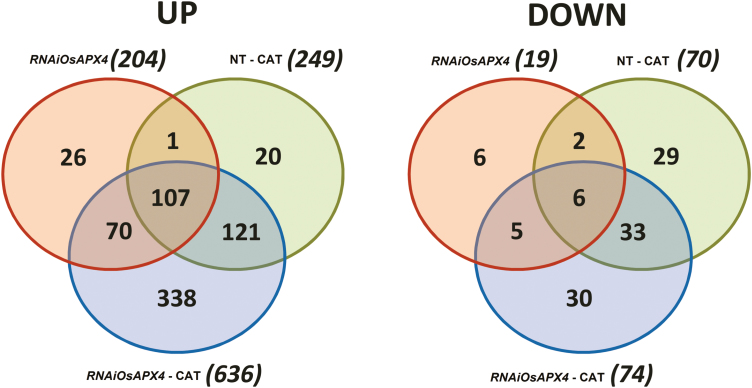
Venn diagrams showing the number of proteins with increased (up) and decreased (down) abundance in leaves of *OsAPX4*-RNAi plants, and *OsAPX4*-RNAi and non-transformed (NT) plants treated with 10 mM of the catalase-inhibitor 3-AT (–CAT), all compared to the protein abundances in NT control plants. Each protein content was considered as being increased if the log_2_ value was higher than 0.5, and decreased if the log_2_ value was lower than –0.5. The diagram was constructed using the Pangloss Venn Diagram Generator (http://www.pangloss.com/seidel/Protocols/venn.cgi).

Summaries of the changes in protein abundance across the treatments are shown in (Fig. 6). There were 70 proteins with increased abundance that were common in both *OsAPX4*-RNAi and *OsAPX4*-RNAi plus CAT-inhibition plants. Among the 636 proteins that had increased abundance in *OsAPX4*-RNAi in the presence of CAT inhibition, only 121 (19%) were also increased in NT plants with CAT inhibition. These results indicated that most of the proteins with increased abundance in the *OsAPX4*-RNAi line subjected to CAT inhibition were not increased in the corresponding NT plants.

### *OsAPX4*-RNAi plants with CAT inhibition exhibit increased amounts of key proteins involved in photosynthesis, redox metabolism, respiration, abiotic stress, and photorespiration

**Fig. 7.**
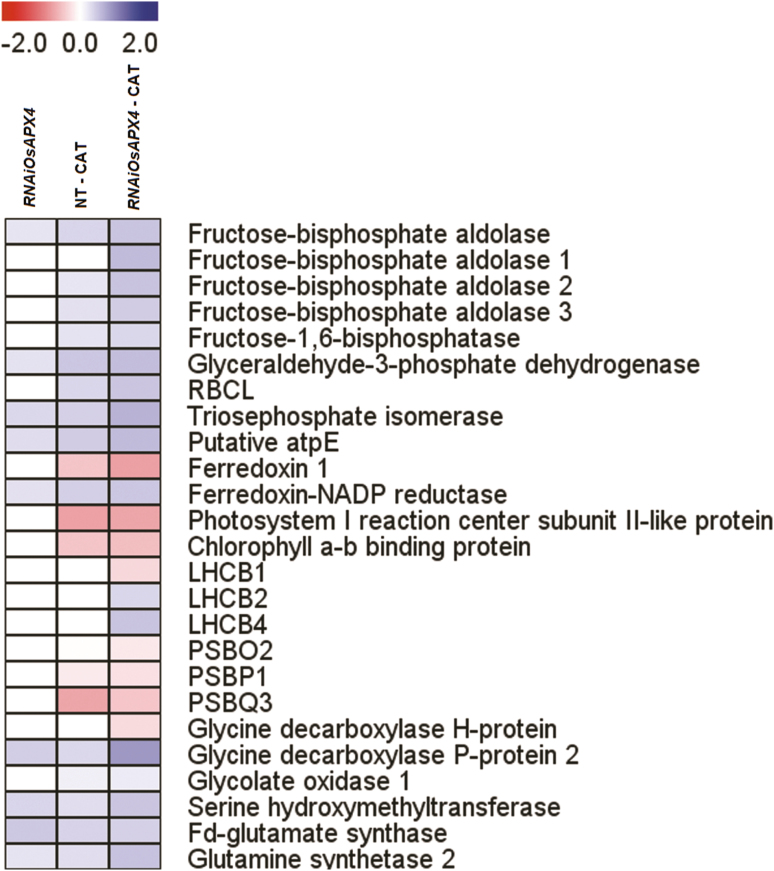
Heatmap of differently expressed proteins related to photosynthetic metabolism in leaves of *OsAPX4*-RNAi plants, and in *OsAPX4*-RNAi and non-transformed (NT) plants treated with 10 mM of the catalase-inhibitor 3-AT (–CAT), all compared to the protein abundances in NT control plants. The fold-changes as assessed by proteomic analysis were plotted as a heatmap using the MEV software framework. The scale is based on a range from –2 to +2 on a log_2_ scale.

*OsAPX4*-RNAi plants without 3-AT treatment displayed significant increases in abundance of the following photosynthesis-related proteins: fructose-biphosphate aldolase, glyceraldehyde-3-phosphate dehydrogenase, triose-phosphate isomerase, and ferredoxin-NADP reductase (Fig. 7). In addition to these proteins, silenced plants under CAT inhibition also exhibited strong increases in abundances of other important photosynthetic proteins, especially those belonging to the Calvin–Benson cycle, such as fructose-biphosphate aldolase isoforms, fructose-1-6-biphosphatase, and Rubisco large subunit. The abundances of glyceraldehyde 3-phosphate dehydrogenase and triose-phosphate isomerase were also much greater than in silenced plants without CAT inhibition. Abundances of other proteins belonging to the photochemical phase were increased: the putative E subunit of chloroplastic ATPase, ferredoxin-NADP reductase (FNR), and the light-harvesting complexes LHCB2 and LHCB4. The abundances of all these proteins except fructose-biphosphate aldolase 1, LHCB2, and LHCB4 were also increased in NT plants under CAT inhibition, but to a lesser extent compared to *OsAPX4*-RNAi (Fig. 7). On the other hand, CAT inhibition also induced reductions in the amount of some proteins involved in the photochemical phase in both NT and *OsAPX4*-RNAi plants, such as ferredoxin 1, PSI reaction center subunit II, chlorophyll *a*-*b* binding protein, PSBO2, PSBP1, and PSBQ3. CAT-inhibited *OsAPX4*-RNAi plants also displayed a decrease in the amount of LHCB1. *OsAPX4*-RNAi plants showed increased amounts of some photorespiratory proteins, including glycine decarboxylase (GDC) P, serine hydroxy-methyl transferase (SHMT), chloroplastic gluthamine synthetase (GS2), and ferredoxin-dependent glutamate synthase (Fd-GOGAT). Under CAT inhibition, these silenced plants showed significant increases in the amounts of GDC, SHMT, and GS2, whereas NT plants under CAT inhibition showed decreases in the amounts of these proteins relative to the silenced plants (Fig. 7).

**Fig. 8.**
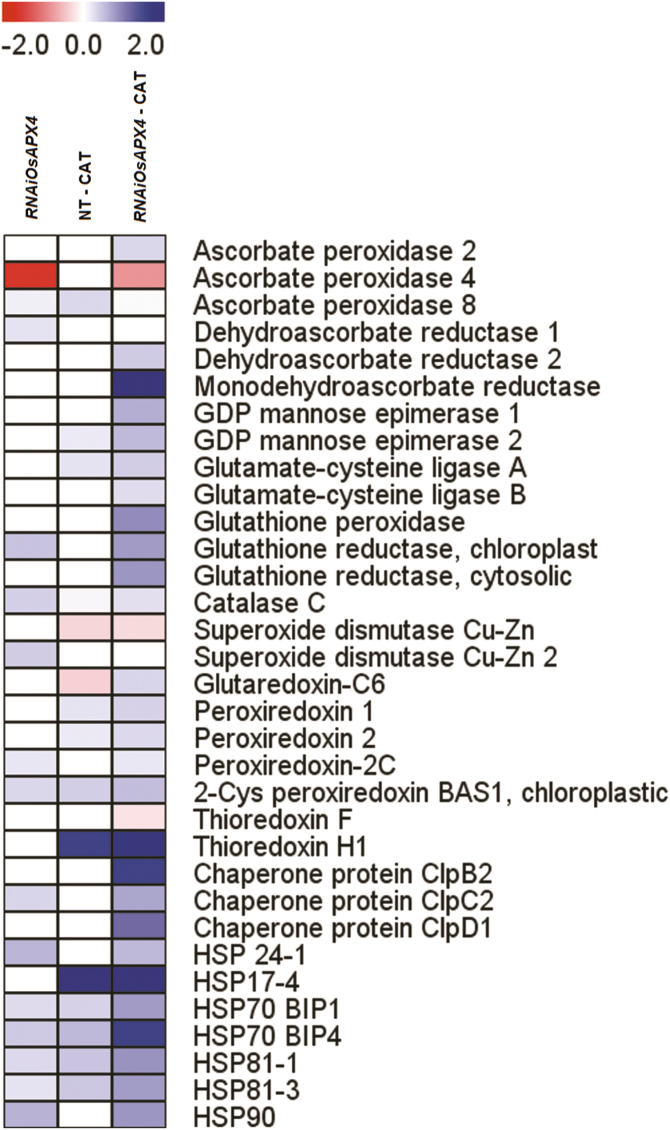
Heatmap of differently expressed proteins related to stress metabolism in leaves of *OsAPX4*-RNAi plants, and in *OsAPX4*-RNAi and non-transformed (NT) plants treated with 10 mM of the catalase-inhibitor 3-AT (–CAT), all compared to the protein abundances in NT control plants. The fold-changes as assessed by proteomic analysis were plotted as a heatmap using the MEV software framework. The scale is based on a range from –2 to +2 on a log_2_ scale.

The responses of proteins involved with redox metabolism and stress defence are shown in Fig. 8. *OsAPX4*-RNAi plants had increased abundance in some important proteins in this group, especially chloroplastic glutathione reductase (GR), superoxide dismutase Cu-Zn 2, 2-Cys peroxiredoxin, chaperone ClpC2, and heat-shock proteins HSP 24-1 and HSP 90. Remarkably, CAT inhibition in *OsAPX4*-RNAi plants induced a strong increase in abundance of most of the proteins belonging to redox defence relative to CAT-inhibited NT plants. Some of these proteins are involved in the ASC–GSH cycle, such as monodehydroascorbate reductase (MDHR), dehydroascorbate reductase (DHR), GR, APX, and glutathione peroxidase (GPX), and in the ASC and GSH synthesis pathways (GDP-mannose epimerases and glutamate-cysteine ligases, respectively), while others are classified as thiol proteins (glutaredoxin, GRX; peroxiredoxins, PRX; and thioredoxin H), chaperone isoforms, and heat-shock proteins (HSP 24-1, HSP 17-4, HSP 70, HSP 81. and HSP 90). Interestingly, most of those redox proteins are targeted to the cytosol and chloroplasts, and none of them are targeted to peroxisomes.

### CAT inhibition in *OsAPX4*-RNAi plants positively regulates proteins related to signalling pathways and to metabolism of nucleic acids, proteins, sugars, and amino acids

*OsAPX4*-RNAi plants showed increases in abundance of several proteins involved in protein synthesis/degradation (Supplementary Fig. S5). Among these, some had strong increases abundance, such as puromycin-sensitive aminopepidase, aspartic proteinase, 26S proteasome regulatory ATPase subunits 4 and 6, and ribosomal proteins 40S, 50S, and 60S. After CAT inhibition, *OsAPX4*-RNAi plants displayed much higher increases in the amounts of several proteins compared to the other treatments, among which were aminopetidase M1, proteasomes, chaperonins, elongations factors, ribosomal proteins 40S, 50S, and 60S, and importin subunits. *OsAPX4*-RNAi plants also showed increases in some proteins related to metabolism of the nucleus, such as histones, putative polyribonucleotide-nucleotidyltransferase, eukaryotic initiation factors, adenylsuccinate synthetase 2, and DEAD-box ATP-dependent RNA helicase 3 and 37 (Supplementary Fig. S6).

*OsAPX4*-RNAi plants grown under normal conditions did not display any changes in the amounts of identified signalling proteins, but their abundances were strongly increased under CAT inhibition (Supplementary Fig. S7). These proteins included 14-3-3-like proteins, calmodulin-like protein 1, calreticulin, ethylene-responsive small GTP-binding protein, a guanine nucleotide-binding protein subunit, and Obg-like ATPase 1. The APX4 deficiency alone also did not affect the amount of proteins involved in amino acid and sugar metabolism (Supplementary Fig. S8); however, the amounts of several of these proteins were greatly increased after CAT inhibition, including some important enzymes such as tryptophan synthase, aspartate aminotransferase, glutamate decarboxylase, glutamine synthetase, 4-alpha-glucanotransferase, fructokinase, sucrose synthases, and sucrose phosphatase. In contrast, very few of these proteins were identified in NT plants subjected to CAT inhibition, and those that were showed only slightly increased amounts.

The overall pattern of accumulation of respiratory proteins was similar to those of the other metabolic classes (Supplementary Fig. S9). Silenced plants without CAT inhibition had accumulation of important enzymes, especially glyceraldehyde-3-phospahate dehydrogenase 1, cytosolic malate dehydrogenase, isocitrate dehydrogenase, pyruvate dehydrogenase, and ATP synthase beta subunit. After CAT inhibition, several other proteins were accumulated in *OsAPX4*-RNAi plants, including enolases, glucose 6-phosphate isomerase, ATP-citrate synthase, aconitase hydratase, dihydrolipoyl dehydrogenase, and succinate-CoA ligase.

## Discussion

We have previously determined that rice *OsAPX4*-RNAi silenced plants display redox changes associated with a higher photosynthetic capacity after pharmacological inhibition of CAT (Sousa *et al.*, 2015). These responses are unexpected since simultaneous deficiency in these peroxisomal peroxidases should induce a more intense oxidative stress due to higher peroxisomal H_2_O_2_ accumulation, and consequently a decreased photosynthetic capacity. However, the underlying metabolic and molecular mechanisms involved with these responses were not addressed in our previous study, nor have they been examined in other reports. Here, we used a combined proteomics and physiological approach in order to examine the underlying mechanisms. Our results showed that some heat-shock proteins were increased in *OsAPX4*-RNAi plants (Fig. 8) and this response suggests that these changes could have been mediated by H_2_O_2_ signalling. Notably, several proteins involved with metabolism of the nucleus (Supplementary Fig. S6) and protein synthesis/degradation (Supplementary Fig. S5) were greatly increased, suggesting that these processes could also have been up-regulated by retrograde H_2_O_2_ signalling.

Several studies employing CAT-deficient plants, especially the Arabidopsis *cat2* mutant, have established that increased peroxisomal H_2_O_2_ is able to trigger intense retrograde signalling (Vandenabeele *et al.*, 2004). However, direct measurement of H_2_O_2_ in plant cells is been challenging because of its short half-life combined with the technical limitations in accessing it in specific cell compartments (Noctor *et al.*, 2016), Although some special molecular tools have been developed, such as the HyPer2 biosensor (Exposito-Rodriguez *et al.*, 2017). This problem is particularly important for detection of H_2_O_2_ originating from pAPX deficiency since this enzyme is bound externally to peroxisome membranes and faces out toward the cytosol (Ribeiro *et al.*, 2017). Nevertheless, our group has recently demonstrated that rice plants deficient in this enzyme display early leaf senescence induced by ROS signalling (Ribeiro *et al.*, 2017), indicating that H_2_O_2_ is probably accumulated in these plants and/or pAPX activity could mediate these responses.

Several reports in the literature have supported the idea that H_2_O_2_ generated in different organelles might be able to migrate to the cytosol and other neighbour organelles via membranes and hence it might reach nucleus, where it could trigger specific signals for gene expression (Mubarakshina *et al.*, 2010; Corpas, 2015; Exposito-Rodriguez *et al.*, 2017; Foyer, 2018). Transcriptomic analyses in Arabidopsis exhibiting differential accumulation of H_2_O_2_ derived from different organelles has demonstrated that it is involved in complex signalling pathways that are dependent on the production site within the cell (Sewelam *et al.* 2014). Thus, H_2_O_2_ specifically produced in peroxisomes should be able to trigger differential gene expression by retrograde signalling (Sewelam *et al.*, 2014). Hence, the original increased H_2_O_2_ signal resulting from pAPX silencing in rice plants, when combined with new ROS signalling resulting from CAT inhibition, could have generated a completely new signal. This new signalling event was then probably associated with the subsequent changes in the proteomic profile that were observed.

**Fig. 9.**
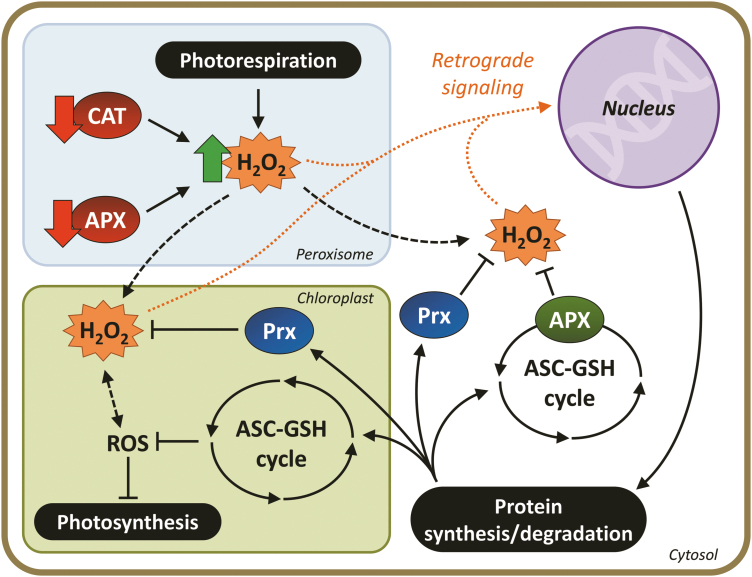
Hypothetical model proposing peroxisomal H_2_O_2_ retrograde signalling after APX4 knockdown followed by catalase (CAT) inhibition in rice leaves. Differential H_2_O_2_ accumulation in peroxisomes of APX-silenced and non-transformed leaves could induce different H_2_O_2_ fluxes towards the cytosol and other compartments, triggering distinct signalling mechanisms. In these cellular compartments (cytosol, chloroplasts, and nucleus), H_2_O_2_ could trigger signalling for increasing synthesis/degradation of several different proteins. It is postulated that the proteins of primary importance for mitigation of the adverse effects induced by CAT inhibition are those belonging to the cytosolic and chloroplast ASC–GSH cycle and other important antioxidant and protective proteins.

Assuming this is a plausible cause–effect relationship to explain the physiological responses that we have previously reported, we propose a general schematic model based on the total proteomic responses obtained in the current study (Fig. 9). This hypothetical model suggests that the burst of H_2_O_2_ initially generated in the peroxisomes due to deficiency in pAPX followed by CAT inhibition is able to trigger a generalized signalling mechanism that reaches various important cellular compartments by a cross-talk mechanism. This assumption is supported by several studies that have employed CAT-deficient plants that suggest that peroxisomal H_2_O_2_ is able to reach other cellular compartments and trigger retrograde signalling (Vandenabeele *et al.*, 2004; Vanderauwera *et al.*, 2011; Corpas, 2015). There is some evidence to suggest that this process might affect different crucial metabolic processes, including gene expression and DNA integrity in the nucleus (Vanderauwera *et al.*, 2011). Thus, we have assumed here that the considerable alterations in the proteomic profile of *OsAPX4*-RNAi plants could have been a consequence of an initial peroxisomal accumulation of H_2_O_2_.

Ascorbate peroxidases have been implicated as important ‘hubs’ in the regulatory networks of H_2_O_2_-dependent signalling processes in higher plants under abiotic stress conditions (Rizhsky *et al.*, 2002; Davletova *et al.*, 2005; Miller *et al.*, 2007; Bonifacio *et al.*, 2011). Remarkably, Bonifacio *et al.* (2016) reported a paradoxical response in rice plants deficient in both cytosolic APX and catalase. The authors suggested that a priming effect induced by increased H_2_O_2_ could have favoured phenotypic plasticity under oxidative stress generated by CAT inhibition. However, the physiological differences that they observed in plants deficient in both cytosolic APX and CAT were much smaller than those that we found in our current study with *OsAPX4*-RNAi plants. Indeed, the large number of differentially expressed proteins exhibited by *OsAPX4*-RNAi plants (Supplementary Tables S1–S3) suggested that the metabolic networks in these plants were strongly affected. Hence, the interaction of these changes with new H_2_O_2_/ROS or other derived redox signals produced after CAT inhibition must have generated a completely new metabolic arrangement, which culminated in the contrasting responses exhibited by NT and *OsAPX4*-RNAi plants.

We consider that the metabolic network in *OsAPX4*-RNAi plants is more acclimated to high levels of peroxisomal H_2_O_2_ as it displayed deeper physiological changes, which could have mitigated against the impairment of photosynthesis in the presence of CAT inhibition. Indeed, all the photosynthetic physiological indicators that we examined clearly showed that *OsAPX4*-RNAi plants displayed much higher photosynthetic efficiency than NT plants under CAT inhibition (Figs 3–5, Supplementary Fig. S2). Silenced plants presented higher maximum carboxylation activity and quantum efficiency of PSII in comparison to NT plants. The essential question therefore is: which are the underlying antioxidant mechanisms displayed by *OsAPX4*-RNAi plants that are able to trigger better photosynthetic performance under CAT inhibition? Several protective mechanisms might have contributed to mitigating the decrease in photosynthesis under these conditions, but an efficient antioxidant system should be the most important of them.

**Fig. 10.**
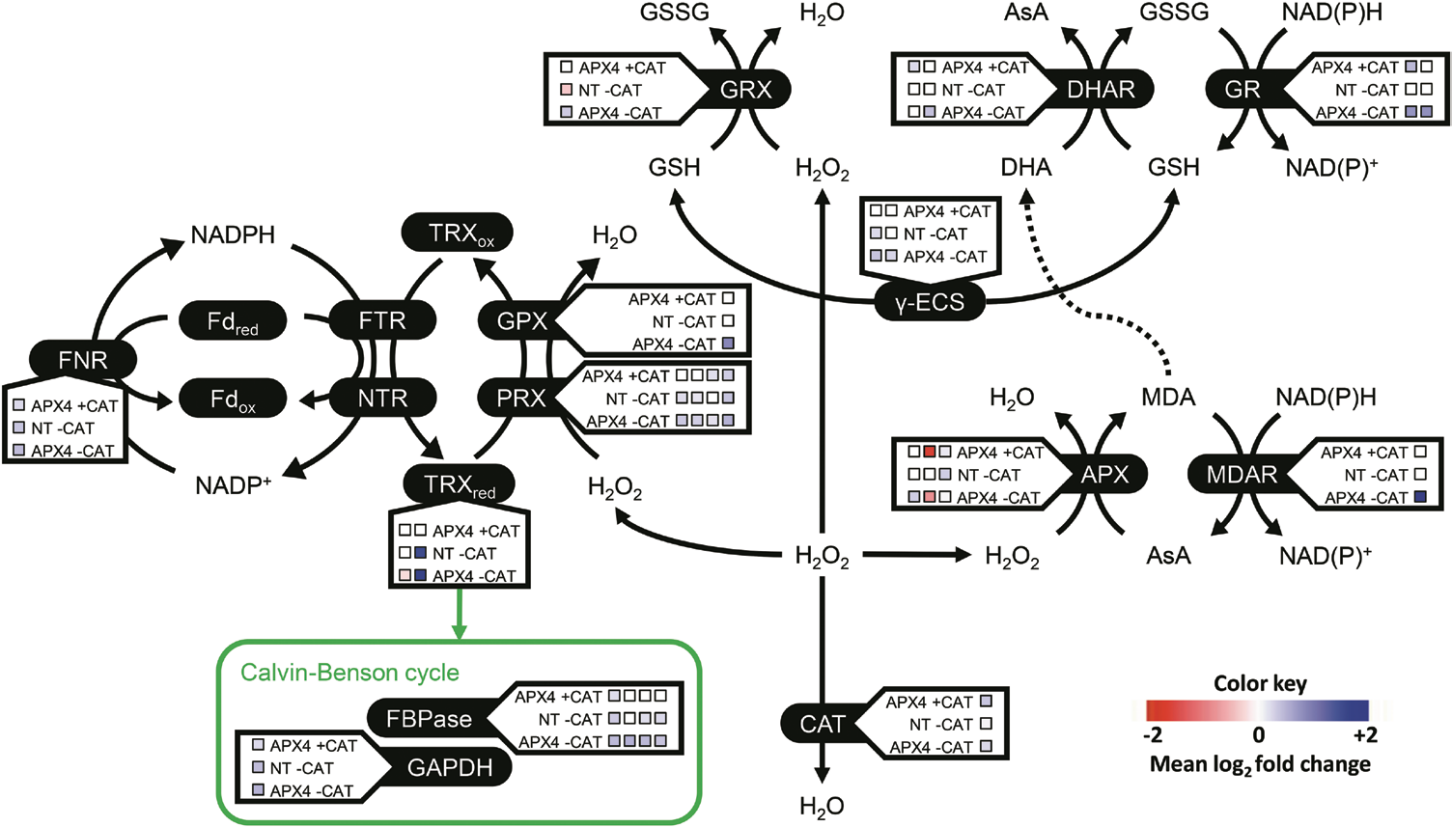
**A** simplified scheme for redox proteins differentially accumulated in non-transformed (NT) and *OsAPX4*-RNAi plants before and after catalase (CAT) inhibition by 3-AT. The proposed scheme does not consider the exact localization of the redox proteins in their respective cellular compartments. The coloured squares for each protein represent a specific isoform, as described below. The colour key indicates the protein abundance on a log_2_ scale. The network highlights the increases in the amounts of several redox proteins in the silenced plants after CAT inhibition, suggesting that these increases may contribute to more effective protection against ROS over-accumulation. The higher content of antioxidant proteins may mitigate the impairment of photosynthesis in the APX-silenced plants after CAT inhibition, as compared to CAT-inhibited NT plants. Abbreviations: γ-ECS, glutamate-cysteine ligase (isoforms: A and B); APX, ascorbate peroxidases (isoforms: 2, 4, and 8); CAT, catalase C; DHAR, dehydroascorbate reductase (isoforms: 1 and 2); FBPase, fructose-bisphosphate aldolase (isoforms: unidentified, 1, 2, and 3); Fd, ferredoxin; FNR, ferredoxin-NADP^+^ reductase; FTR, ferredoxin-thioredoxin reductase; GAPDH, glyceraldehyde-3-phosphate dehydrogenase; GPX, glutathione peroxidase; GR, glutathione reductase (chloroplastic and cytosolic); GRX, glutaredoxin-C6; MDAR, monodehydroascorbate reductase; NTR, NADPH-Trx reductase; PRX, peroxiredoxin (isoforms: 1, 2, and 2C, and chloroplastic 2-Cys peroxiredoxin BAS1); TRX, thioredoxin (isoforms: F and H1).

Over-accumulation of ROS in chloroplasts drastically affects photosynthesis via several mechanisms, including PSII photoinhibition due to delays in D1 protein synthesis (Jimbo *et al.*, 2018) and inactivation of Calvin–Benson cycle enzymes (Davletova *et al.*, 2005). To explain the more effective antioxidant protective mechanisms triggered in *OsAPX4*-RNAi plants, we have constructed a redox protein network based on the proteomic responses (Fig. 10). Our hypothesis is that the accumulation of antioxidant-related proteins, especially the components of the ASC–GSH cycle and other related thiol proteins, is probably the most important factor in generating the more effective antioxidant protection for photosynthetic apparatus in the silenced plants. Whilst each differently accumulated protein could have contributed directly or indirectly to the final *OsAPX4*-RNAi phenotype, the presence of a robust compensatory antioxidative system must have been primary in avoiding oxidative stress and its general downstream deleterious effects. Indeed, it is widely known that the presence of an efficient antioxidant system in chloroplasts and the cytosol is crucial for photosynthetic protection in plants (Davletova *et al.*, 2005; Maruta *et al.*, 2010, 2016; Naranjo *et al.*, 2016).

*OsAPX4*-RNAi plants accumulated important antioxidant proteins targeted to the cytosol and chloroplasts, especially those belonging to the ASC–GSH cycle, some important peroxidases, glutaredoxins, thioredoxins, GDP-mannose epimerases (involved in the ASC synthesis pathway), and glutamate-cysteine ligases (the most important enzyme for GSH synthesis) (Fig. 10). It has been widely reported that coordinated action of these enzymes and non-enzymatic reducing ASC and GSH agents contribute to maintenance of H_2_O_2_ homeostasis, the avoidance of oxidative stress, and mitigation of impairment of photosynthesis in the presence of excess ROS (Foyer and Noctor, 2011; Rahantaniaina *et al.*, 2017). Although previous work by our group has shown a slight decrease in ASC content in *OsAPX4*-RNAi plants (Sousa *et al.*, 2015), usually the concentration of this antioxidant found in leaves is not limiting for APX activity, as we have recently demonstrated in rice (Castro *et al.*, 2018). Interestingly, NT and *OsAPX4*-RNAi plants under CAT inhibition did not show any changes in the amounts of any antioxidant proteins targeted to the peroxisomes (Fig. 8). However, this does not discount the possibility that some peroxisomal redox proteins may have an important role in the silenced plants since the proteomic analysis was limited in its ability to consider proteins localized in these organelles. Taken together, these findings suggest that antioxidant systems localized in the cytosol and chloroplasts, especially the ASC–GSH cycle, are important for coping with excess peroxisomal H_2_O_2_.

Interestingly, two glutamate-cysteine ligases, the most important enzyme of the GSH synthesis pathway, were strongly accumulated in *OsAPX4*-RNAi plants under CAT inhibition (Fig. 8). We have previously demonstrated that rice plants under CAT inhibition display a notable increase in both the total glutathione pool and GSH oxidation state, suggesting that GSH redox changes could be also involved in the signalling process, alone or in addition to peroxisomal H_2_O_2_ (Sousa *et al.*, 2015; Bonifacio *et al.*, 2016). In parallel, these responses are associated with up-regulation of genes encoding for both the cytosolic APX isoforms (Sousa *et al.*, 2015; Bonifacio *et al.*, 2016) and for APX2 accumulation, as found in the present study (Fig. 8), demonstrating that H_2_O_2_ accumulated in peroxisomes is able to reach the cytosol and to display signalling responses via the cytosolic APX system, possibly involving the ASC–GSH cycle (Davletova *et al.*, 2005). However, this possibility does not rule out an overlapping mechanism involving a combined signalling pathway initiated by H_2_O_2_ and GSH (Gao *et al.*, 2014; König *et al.*, 2017).

In summary, we have determined some of the mechanisms by which rice plants deficient in pAPX and subjected to CAT inhibition exhibit higher photosynthetic resilience under oxidative stress compared to non-transformed plants. Interestingly, these plants also presented higher abundances of important photosynthetic and photorespiratory proteins, which might have positively contributed towards energy use efficiency in photosynthesis. They also benefited from up-regulation of several antioxidant and protective proteins, which contributed to an improved ROS balance in chloroplasts. These results reinforce the importance of the balance of peroxisomal H_2_O_2_ in the regulation of the expression of genes encoding for these proteins. Thus, this study demonstrates for the first time that pAPX knockdown followed by CAT inhibition results in metabolic changes that significantly affect several networks involved in photosynthetic performance under adverse conditions of oxidative stress. These findings are of practical importance since high photorespiration in presence of CAT deficiency is a common situation in field conditions for C_3_ plants, especially under abiotic stress such as excess light.

## Supplementary data

Supplementary data are available at *JXB* online.

Table S1. Differentially expressed proteins in leaves of *OsAPX4*-RNAi under normal growth conditions compared to non-transformed control plants.

Table S2. Differentially expressed proteins in leaves of non-transformed plants with CAT inhibition compared to control plants.

Table S3. Differentially expressed proteins in leaves of *OsAPX4*-RNAi plants with CAT inhibition compared to non-transformed control plants.

Fig. S1. Images of non-transformed and *OSAPX4*-RNAi plants with and without CAT inhibition.

Fig. S2. *A*/*C*_i_ curves in non-transformed and *OsAPX4*-RNAi plants with and without CAT inhibition.

Fig. S3. Functional classification of proteins with increased abundance relative to non-transformed control plants.

Fig. S4. Functional classification of proteins with decreased abundance relative to non-transformed control plants.

Fig. S5. Heatmap of differentially expressed proteins in leaves related to protein metabolism relative to non-transformed control plants.

Fig. S6. Heatmap of differentially expressed proteins in leaves related to metabolism of the nucleus relative to non-transformed control plants.

Fig. S7. Heatmap of differentially expressed proteins in leaves related to signalling relative to non-transformed control plants.

Fig. S8. Heatmap of differently expressed proteins in leaves related to amino acids and sugar metabolism relative to non-transformed control plants.

## Supplementary Material

Supplementary Table S1

Supplementary Table S2

Supplementary Table S3

Supplementary Figures

## References

[CIT0001] Bonifacio A, Carvalho FEL, Martins MO, Lima Neto MC, Cunha JR, Ribeiro CW, Margis-Pinheiro M, Silveira JAG 2016 Silenced rice in both cytosolic ascorbate peroxidases displays pre-acclimation to cope with oxidative stress induced by 3-aminotriazole-inhibited catalase. Journal of Plant Physiology 201, 17–27. 27379617 10.1016/j.jplph.2016.06.015

[CIT0002] Bonifacio A, Martins MO, Ribeiro CW, Fontenele AV, Carvalho FE, Margis-Pinheiro M, Silveira JA 2011 Role of peroxidases in the compensation of cytosolic ascorbate peroxidase knockdown in rice plants under abiotic stress. Plant, Cell & Environment 34, 1705–1722. 10.1111/j.1365-3040.2011.02366.x 21631533

[CIT0003] Bradford MM 1976 A rapid and sensitive method for the quantitation of microgram quantities of protein utilizing the principle of protein-dye binding. Analytical Biochemistry 72, 248–254. 942051 10.1016/0003-2697(76)90527-3

[CIT0004] Castro JLS, Lima-Melo Y, Carvalho FEL, Feitosa AGS, Lima Neto MC, Caverzan A, Margis-Pinheiro M, Silveira JAG 2018 Ascorbic acid toxicity is related to oxidative stress and enhanced by high light and knockdown of chloroplast ascorbate peroxidases in rice plants. Theoretical and Experimental Plant Physiology 30, 41–55.

[CIT0005] Chamnongpol S, Willekens H, Langebartels C, van Montagu M, Inze D, Van Camp W 1996 Transgenic tobacco with a reduced catalase activity develops necrotic lesions and induces pathogenesis-related expression under high light. The Plant Journal 10, 491–503.

[CIT0006] Corpas FJ 2015 What is the role of hydrogen peroxide in plant peroxisomes?Plant Biology 17, 1099–1103. 26242708 10.1111/plb.12376

[CIT0007] Davletova S, Rizhsky L, Liang H, Shengqiang Z, Oliver DJ, Coutu J, Shulaev V, Schlauch K, Mittler R 2005 Cytosolic ascorbate peroxidase 1 is a central component of the reactive oxygen gene network of Arabidopsis. The Plant Cell 17, 268–281. 15608336 10.1105/tpc.104.026971 PMC544504

[CIT0008] Exposito-Rodriguez M, Laissue PP, Yvon-Durocher G, Smirnoff N, Mullineaux PM 2017 Photosynthesis-dependent H_2_O_2_ transfer from chloroplasts to nuclei provides a high-light signalling mechanism. Nature Communications 8, 49. 10.1038/s41467-017-00074-w PMC5491514 28663550

[CIT0009] Foyer CH 2018 Reactive oxygen species, oxidative signaling and the regulation of photosynthesis. Environmental and Experimental Botany 154, 134–142. 30283160 10.1016/j.envexpbot.2018.05.003 PMC6105748

[CIT0010] Foyer CH, Noctor G 2011 Ascorbate and glutathione: the heart of the redox hub. Plant Physiology 155, 2–18. 21205630 10.1104/pp.110.167569 PMC3075780

[CIT0011] Gao X, Yuan HM, Hu YQ, Li J, Lu YT 2014 Mutation of Arabidopsis *CATALASE2* results in hyponastic leaves by changes of auxin levels. Plant, Cell & Environment 37, 175–188. 10.1111/pce.12144 23738953

[CIT0012] Han Y, Chaouch S, Mhamdi A, Queval G, Zechmann B, Noctor G 2013 Functional analysis of Arabidopsis mutants points to novel roles for glutathione in coupling H_2_O_2_ to activation of salicylic acid accumulation and signaling. Antioxidants & Redox Signaling 18, 2106–2121. 23148658 10.1089/ars.2012.5052 PMC3629853

[CIT0013] Havir EA, McHale NA 1987 Biochemical and developmental characterization of multiple forms of catalase in tobacco leaves. Plant Physiology 84, 450–455. 16665461 10.1104/pp.84.2.450 PMC1056601

[CIT0014] Hertwig B, Streb P, Feierabend J 1992 Light dependence of catalase synthesis and degradation in leaves and the influence of interfering stress conditions. Plant Physiology 100, 1547–1553. 16653156 10.1104/pp.100.3.1547 PMC1075818

[CIT0015] Hoagland DR, Arnon DI 1950 The water-culture method for growing plants without soil. California Agricultural Experiment Station Circular 347, 1–32.

[CIT0016] Jimbo H, Yutthanasirikul R, Nagano T, Hisabori T, Hihara Y, Nishiyama Y 2018 Oxidation of translation factor EF-Tu inhibits the repair of photosystem II. Plant Physiology 176, 2691–2699. 29439212 10.1104/pp.18.00037 PMC5884602

[CIT0017] Kavitha K, Venkataraman G, Parida A 2008 An oxidative and salinity stress induced peroxisomal ascorbate peroxidase from *Avicennia marina*: molecular and functional characterization. Plant Physiology and Biochemistry 46, 794–804. 18614374 10.1016/j.plaphy.2008.05.008

[CIT0018] König K, Vaseghi MJ, Dreyer A, Dietz K-J 2017 The significance of glutathione and ascorbate in modulating the retrograde high light response in *Arabidopsis thaliana* leaves. Physiologia Plantarum 176, 2691–2699. 10.1111/ppl.12644 28984358

[CIT0019] Maruta T, Sawa Y, Shigeoka S, Ishikawa T 2016 Diversity and evolution of ascorbate peroxidase functions in chloroplasts: more than just a classical antioxidant enzyme?Plant & Cell Physiology 57, 1377–1386. 26738546 10.1093/pcp/pcv203

[CIT0020] Maruta T, Tanouchi A, Tamoi M, Yabuta Y, Yoshimura K, Ishikawa T, Shigeoka S 2010 Arabidopsis chloroplastic ascorbate peroxidase isoenzymes play a dual role in photoprotection and gene regulation under photooxidative stress. Plant & Cell Physiology 51, 190–200. 20007290 10.1093/pcp/pcp177

[CIT0021] Mhamdi A, Noctor G, Baker A 2012 Plant catalases: peroxisomal redox guardians. Archives of Biochemistry and Biophysics 525, 181–194. 22546508 10.1016/j.abb.2012.04.015

[CIT0022] Miller G, Suzuki N, Rizhsky L, Hegie A, Koussevitzky S, Mittler R 2007 Double mutants deficient in cytosolic and thylakoid ascorbate peroxidase reveal a complex mode of interaction between reactive oxygen species, plant development, and response to abiotic stresses. Plant Physiology 144, 1777–1785. 17556505 10.1104/pp.107.101436 PMC1949877

[CIT0023] Mubarakshina MM, Ivanov BN, Naydov IA, Hillier W, Badger MR, Krieger-Liszkay A 2010 Production and diffusion of chloroplastic H_2_O_2_ and its implication to signalling. Journal of Experimental Botany 61, 3577–3587. 20595239 10.1093/jxb/erq171

[CIT0024] Mullen RT, Lisenbee CS, Miernyk JA, Trelease RN 1999 Peroxisomal membrane ascorbate peroxidase is sorted to a membranous network that resembles a subdomain of the endoplasmic reticulum. The Plant Cell 11, 2167–2185. 10559442 10.1105/tpc.11.11.2167 PMC144122

[CIT0025] Naranjo B, Diaz-Espejo A, Lindahl M, Cejudo FJ 2016 Type-*f* thioredoxins have a role in the short-term activation of carbon metabolism and their loss affects growth under short-day conditions in *Arabidopsis thaliana*. Journal of Experimental Botany 67, 1951–1964. 26842981 10.1093/jxb/erw017 PMC4783373

[CIT0026] Narendra S, Venkataramani S, Shen G, Wang J, Pasapula V, Lin Y, Kornyeyev D, Holaday AS, Zhang H 2006 The Arabidopsis ascorbate peroxidase 3 is a peroxisomal membrane-bound antioxidant enzyme and is dispensable for Arabidopsis growth and development. Journal of Experimental Botany 57, 3033–3042. 16873450 10.1093/jxb/erl060

[CIT0027] Noctor G, Mhamdi A, Foyer CH 2016 Oxidative stress and antioxidative systems: recipes for successful data collection and interpretation. Plant, Cell & Environment 39, 1140–1160. 10.1111/pce.12726 26864619

[CIT0028] Nyathi Y, Baker A 2006 Plant peroxisomes as a source of signalling molecules. Biochimica et Biophysica Acta 1763, 1478–1495. 17030442 10.1016/j.bbamcr.2006.08.031

[CIT0029] Polidoros AN, Scandalios JG 1997 Response of the maize catalases to light. Free Radical Biology & Medicine 23, 497–504. 9214588 10.1016/s0891-5849(97)00110-x

[CIT0030] Rahantaniaina MS, Li S, Chatel-Innocenti G, Tuzet A, Mhamdi A, Vanacker H, Noctor G 2017 Glutathione oxidation in response to intracellular H_2_O_2_: key but overlapping roles for dehydroascorbate reductases. Plant Signaling & Behavior 12, e1356531. 28782990 10.1080/15592324.2017.1356531 PMC5616140

[CIT0031] Ribeiro CW, Korbes AP, Garighan JA, Jardim-Messeder D, Carvalho FEL, Sousa RHV, Caverzan A, Teixeira FK, Silveira JAG, Margis-Pinheiro M 2017 Rice peroxisomal ascorbate peroxidase knockdown affects ROS signaling and triggers early leaf senescence. Plant Science 263, 55–65. 28818384 10.1016/j.plantsci.2017.07.009

[CIT0032] Rizhsky L, Hallak-Herr E, Van Breusegem F, Rachmilevitch S, Barr JE, Rodermel S, Inzé D, Mittler R 2002 Double antisense plants lacking ascorbate peroxidase and catalase are less sensitive to oxidative stress than single antisense plants lacking ascorbate peroxidase or catalase. The Plant Journal 32, 329–342. 12410811 10.1046/j.1365-313x.2002.01427.x

[CIT0033] Schreiber U, Bilger W, Neubauer C 1995 Chlorophyll fluorescence as a nonintrusive indicator for rapid assessment of *in vivo* photosynthesis. In: Schulze E-D, Caldwell MM, eds. Ecophysiology of photosynthesis. Berlin, Heidelberg: Springer, 49–70.

[CIT0034] Sewelam N, Jaspert N, Van Der Kelen K, Tognetti VB, Schmitz J, Frerigmann H, Stahl E, Zeier J, Van Breusegem F, Maurino VG 2014 Spatial H_2_O_2_ signaling specificity: H_2_O_2_ from chloroplasts and peroxisomes modulates the plant transcriptome differentially. Molecular Plant 7, 1191–1210. 24908268 10.1093/mp/ssu070

[CIT0035] Sharkey TD, Bernacchi CJ, Farquhar GD, Singsaas EL 2007 Fitting photosynthetic carbon dioxide response curves for C_3_ leaves. Plant, Cell & Environment 30, 1035–1040. 10.1111/j.1365-3040.2007.01710.x 17661745

[CIT0036] Sousa RH, Carvalho FE, Ribeiro CW, Passaia G, Cunha JR, Lima-Melo Y, Margis-Pinheiro M, Silveira JA 2015 Peroxisomal APX knockdown triggers antioxidant mechanisms favourable for coping with high photorespiratory H_2_O_2_ induced by CAT deficiency in rice. Plant, Cell & Environment 38, 499–513. 10.1111/pce.12409 25039271

[CIT0037] Towbin H, Staehelin T, Gordon J 1979 Electrophoretic transfer of proteins from polyacrylamide gels to nitrocellulose sheets: procedure and some applications. Proceedings of the National Academy of Sciences, USA 76, 4350–4354. 10.1073/pnas.76.9.4350 PMC411572 388439

[CIT0038] Usadel B, Nagel A, Thimm O, et al 2005 Extension of the visualization tool MapMan to allow statistical analysis of arrays, display of corresponding genes, and comparison with known responses. Plant Physiology 138, 1195–1204. 16009995 10.1104/pp.105.060459 PMC1176394

[CIT0039] Vandenabeele S, Vanderauwera S, Vuylsteke M, Rombauts S, Langebartels C, Seidlitz HK, Zabeau M, Van Montagu M, Inzé D, Van Breusegem F 2004 Catalase deficiency drastically affects gene expression induced by high light in *Arabidopsis thaliana*. The Plant Journal 39, 45–58. 15200641 10.1111/j.1365-313X.2004.02105.x

[CIT0040] Vanderauwera S, Suzuki N, Miller G, et al 2011 Extranuclear protection of chromosomal DNA from oxidative stress. Proceedings of the National Academy of Sciences, USA 108, 1711–1716. 10.1073/pnas.1018359108 PMC3029710 21220338

[CIT0041] Vanderauwera S, Zimmermann P, Rombauts S, Vandenabeele S, Langebartels C, Gruissem W, Inzé D, Van Breusegem F 2005 Genome-wide analysis of hydrogen peroxide-regulated gene expression in Arabidopsis reveals a high light-induced transcriptional cluster involved in anthocyanin biosynthesis. Plant Physiology 139, 806–821. 16183842 10.1104/pp.105.065896 PMC1255997

[CIT0042] Voss I, Sunil B, Scheibe R, Raghavendra AS 2013 Emerging concept for the role of photorespiration as an important part of abiotic stress response. Plant Biology 15, 713–722. 23452019 10.1111/j.1438-8677.2012.00710.x

[CIT0043] Wang J, Zhang H, Allen RD 1999 Overexpression of an Arabidopsis peroxisomal ascorbate peroxidase gene in tobacco increases protection against oxidative stress. Plant & Cell Physiology 40, 725–732. 10501032 10.1093/oxfordjournals.pcp.a029599

[CIT0044] Willekens H, Chamnongpol S, Davey M, Schraudner M, Langebartels C, Van Montagu M, Inzé D, Van Camp W 1997 Catalase is a sink for H_2_O_2_ and is indispensable for stress defence in C_3_ plants. The EMBO Journal 16, 4806–4816. 9305623 10.1093/emboj/16.16.4806 PMC1170116

[CIT0045] Xu W-F, Shi W-M, Ueda A, Takabe T 2008 Mechanisms of salt tolerance in transgenic *Arabidopsis thaliana* carrying a peroxisomal ascorbate peroxidase gene from barley. Pedosphere 18, 486–495.

[CIT0046] Yamaguchi K, Mori H, Nishimura M 1995 A novel isoenzyme of ascorbate peroxidase localized on glyoxysomal and leaf peroxisomal membranes in pumpkin. Plant & Cell Physiology 36, 1157–1162. 8528608 10.1093/oxfordjournals.pcp.a078862

[CIT0047] Zhang Y, Wen Z, Washburn MP, Florens L 2009 Effect of dynamic exclusion duration on spectral count based quantitative proteomics. Analytical Chemistry 81, 6317–6326. 19586016 10.1021/ac9004887

