# Supplementary material for: Impairment of peroxisomal APX and CAT activities increases protection of photosynthesis under oxidative stress
Source: J Exp Bot. 2018 Oct 12;70(2):627–39. doi: 10.1093/jxb/ery354 (PMC6322566; doi:10.1093/jxb/ery354)
Supplement: Supplementary Figures [file ery354_suppl_supplementary_figures.pdf]

Impairment of peroxisomal APX and CAT activities increases protection of photosynthesis under oxidative stress conditions

Rachel H.V. Sousa[1], Fabricio E. L. Carvalho[1], Yugo Lima-Melo[1,2], Vicente T.C.B Alencar[1], Danilo M. Daloso[1], Marcia Margis-Pinheiro[3],Setsuko Komatsu[4] & Joaquim A. G. Silveira[1*]

SUPPLAMENTARY MATERIAL

2018

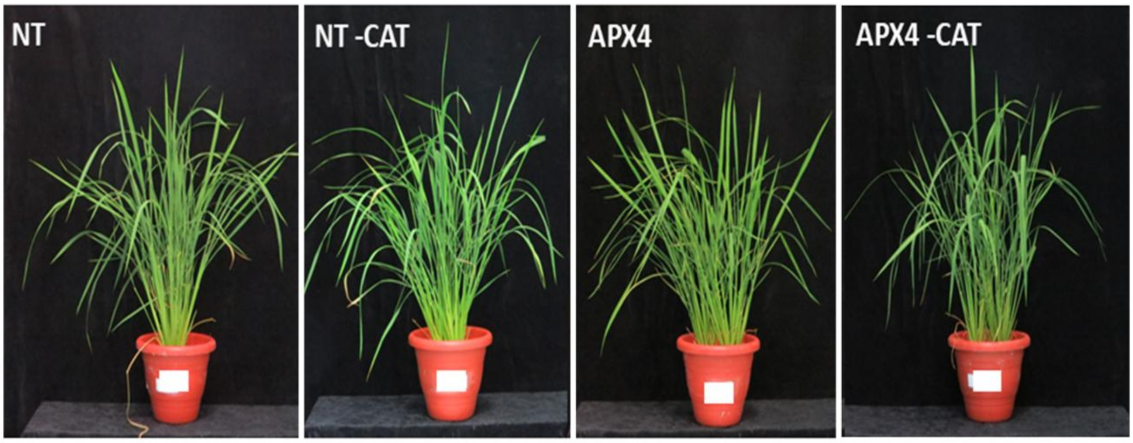

Supplementary figure 1. Morphological aspects of shoots of non-transformed (NT) and *RNAiOsAPX4* rice plants exposed 10 mM 3-AT (-CAT) and control. Plants are the most representative from four biological replicates.

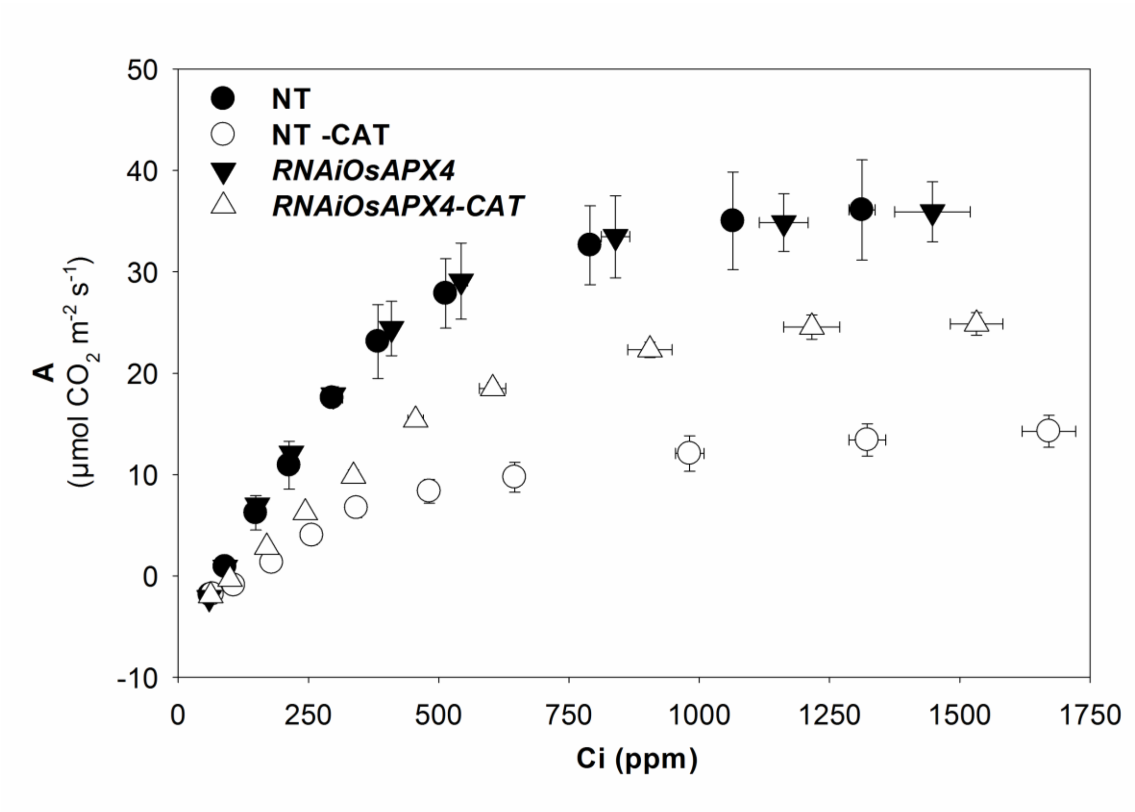

Supplementary figure 2. P_N/Ci curves non-transformed (NT) and *RNAiOsAPX4* rice plants exposed 10 mM 3-AT (-CAT) and control. The points represent means of four replicates ±SD.

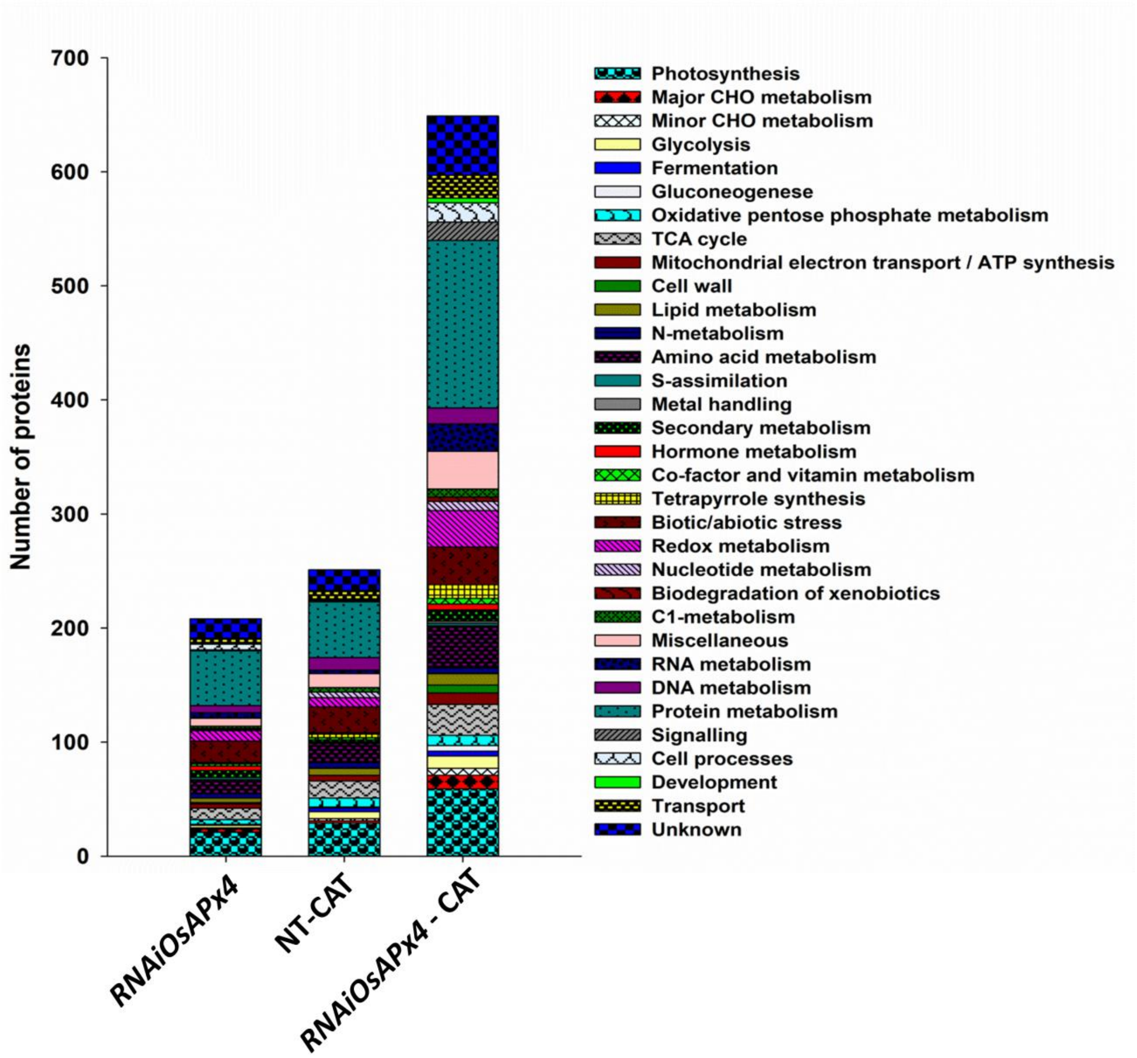

Supplementary figure 3. Functional classification of proteins with increased abundance. The amount of each protein in leaves of *RNAiOsAPX4* under normal growth condition and in non-transformed (NT) and *RNAiOsAPX4* plants exposed to 10 mM 3-AT (-CAT) were all compared to the respective protein amount found in NT control plants. Each protein content was considered increased if the log2 value was higher than 0.5. Proteins were classified by the software (MapMan) according to their involvement in each metabolic process.

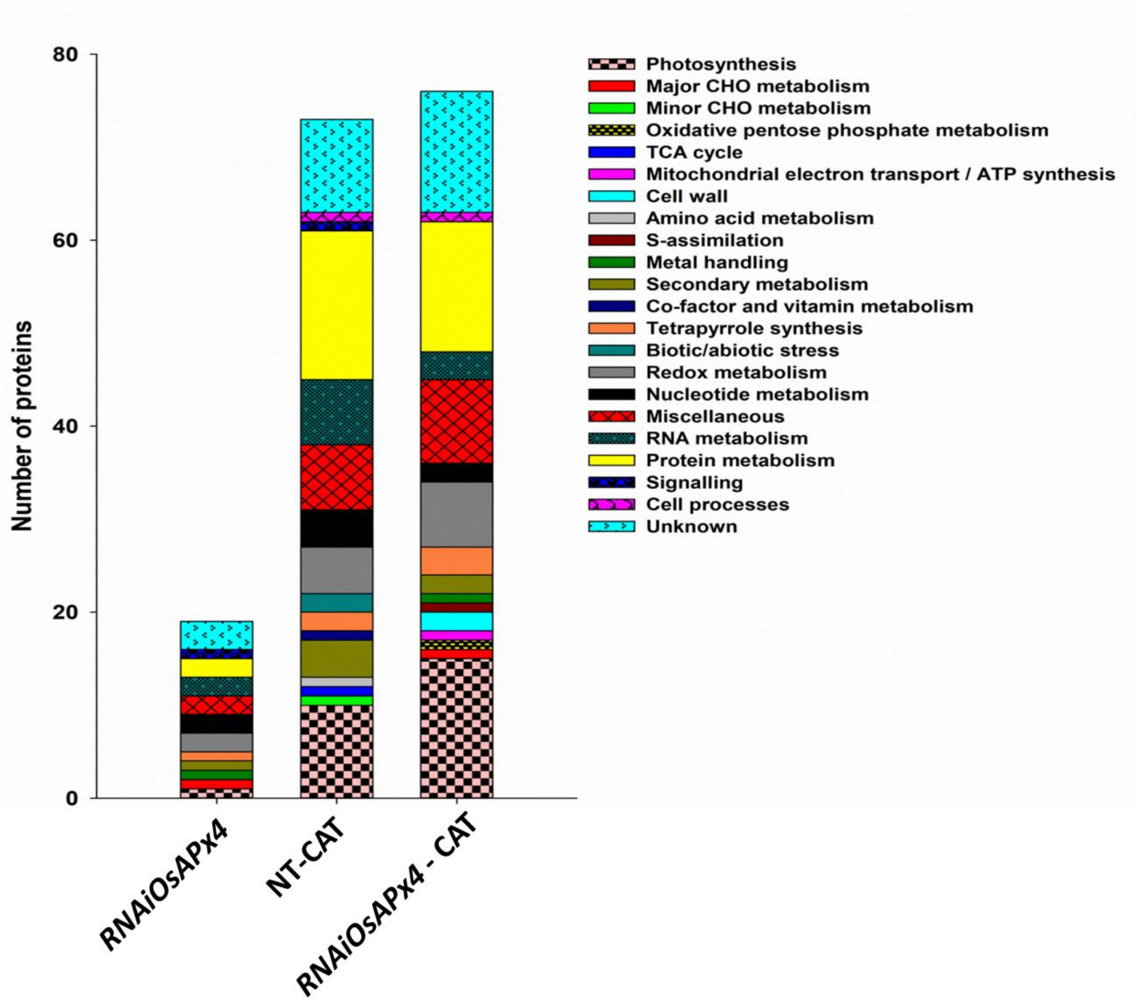

Supplementary figure 4. Functional classification of proteins with decreased abundance. The amount of each protein in leaves of *RNAiOsAPX4* under normal growth condition and in non-transformed (NT) and *RNAiOsAPX4* plants exposed to 10 mM 3-AT (-CAT) were all compared to the respective protein amount found in NT control plants. Each protein content was considered increased if the log2 value was higher than 0.5. Proteins were classified by the software (MapMan) according to their involvement in each metabolic process.

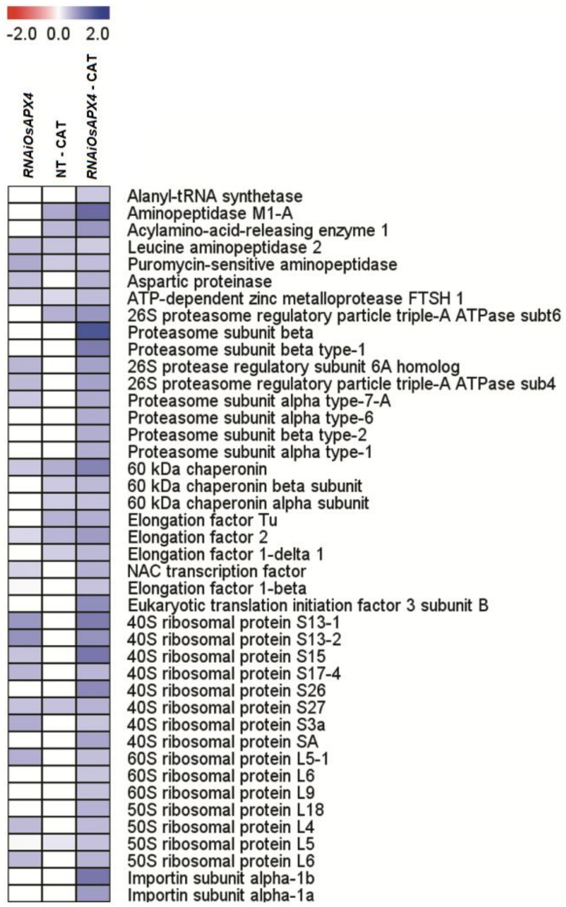

Supplementary figure 5. Heatmap of differently expressed proteins related to protein metabolism in leaves of *RNAiOsAPX4* under normal growth condition and in non-transformed (NT) and *RNAiOsAPX4* plants exposed to 10 mM 3-AT (-CAT), all compared to the respective protein amount in NT control plants.The fold change from proteomics was plotted into a heatmap using MEV software framework.

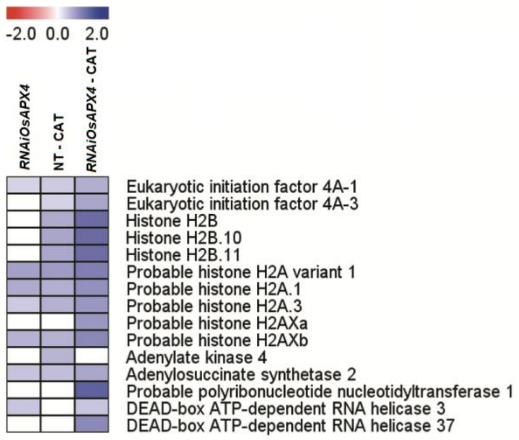

Supplementary figure 6. Heatmap of differently expressed proteins related to nuclear metabolism in leaves of *RNAiOsAPX4* under normal growth condition and in non-transformed (NT) and *RNAiOsAPX4* plants exposed to 10 mM 3-AT (-CAT), all compared to the respective protein amount in NT control plants.The fold change from proteomics was plotted into a heatmap using MEV software framework.

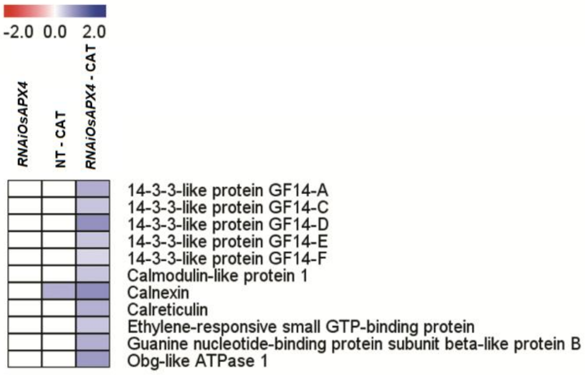

Supplementary figure 7.Heatmap of differently expressed proteins related to signalling metabolism in leaves of *RNAiOsAPX4* under normal growth condition and in non-transformed (NT) and *RNAiOsAPX4* plants exposed to 10 mM 3-AT (-CAT), all compared to the respective protein amount in NT control plants.The fold change from proteomics was plotted into a heatmap using MEV software framework.

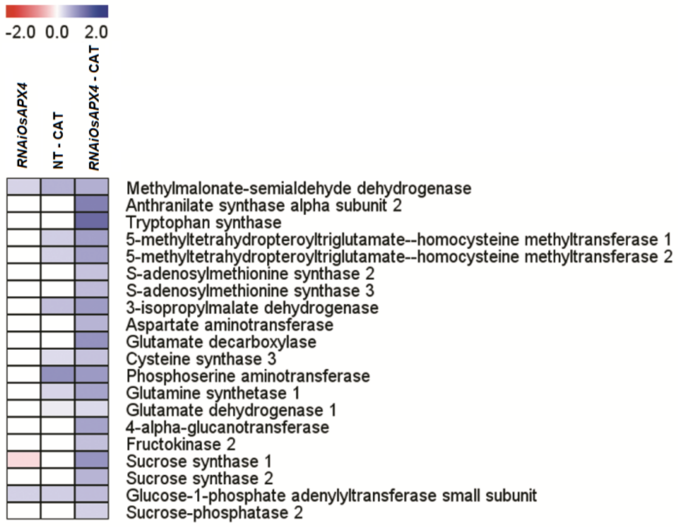

Supplementary figure 8.Heatmap of differently expressed proteins related to amino acid and sugar metabolismsin leaves of *RNAiOsAPX4* under normal growth condition and in non-transformed (NT) and *RNAiOsAPX4* plants exposed to 10 mM 3-AT (-CAT), all compared to the respective protein amount in NT control plants.The fold change from proteomics was plotted into a heatmap using MEV software framework.

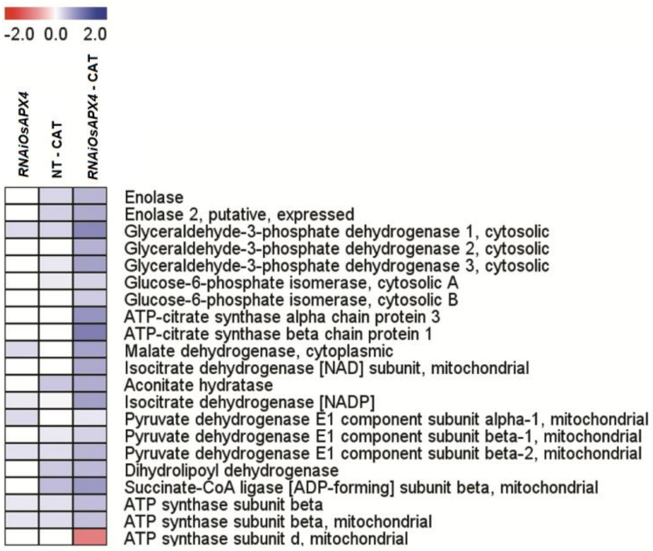

Supplementary figure 9. Heatmap of differently expressed proteins related to respiratory metabolism in leaves of *RNAiOsAPX4* under normal growth condition and in non-transformed (NT) and *RNAiOsAPX4* plants exposed to 10 mM 3-AT (-CAT), all compared to the respective protein amount in NT control plants.The fold change from proteomics was plotted into a heatmap using MEV software framework.
